# The songbird syrinx morphome: a three-dimensional, high-resolution, interactive morphological map of the zebra finch vocal organ

**DOI:** 10.1186/1741-7007-11-1

**Published:** 2013-01-08

**Authors:** Daniel N Düring, Alexander Ziegler, Christopher K Thompson, Andreas Ziegler, Cornelius Faber, Johannes Müller, Constance Scharff, Coen PH Elemans

**Affiliations:** 1 Verhaltensbiologie, Freie Universität Berlin, Takustrasse 6, 14195 Berlin, Germany; 2 Museum of Comparative Zoology, Department of Organismic and Evolutionary Biology, Harvard University, 26 Oxford Street, Cambridge, MA 02138, USA; 3 Current address: Department of Cell Biology, The Scripps Research Institute, 10550 North Torrey Pine Road, La Jolla, CA 92037, USA; 4 Institut für Immungenetik, Charité-Universitätsmedizin Berlin, Freie Universität Berlin, Thielallee 73, 14195 Berlin, Germany; 5 Institut für Klinische Radiologie, Universitätsklinikum Münster, Albert-Schweitzer-Campus 1, 48149 Münster, Germany; 6 Museum für Naturkunde, Leibniz-Institut für Evolutions und Biodiversitätsforschung, Humboldt-Universität zu Berlin, Invalidenstraße 43, 10115 Berlin, Germany; 7 Institute of Biology, University of Southern Denmark, Campusvej 55, 5230 Odense, Denmark

## Abstract

**Background:**

Like human infants, songbirds learn their species-specific vocalizations through imitation learning. The birdsong system has emerged as a widely used experimental animal model for understanding the underlying neural mechanisms responsible for vocal production learning. However, how neural impulses are translated into the precise motor behavior of the complex vocal organ (syrinx) to create song is poorly understood. First and foremost, we lack a detailed understanding of syringeal morphology.

**Results:**

To fill this gap we combined non-invasive (high-field magnetic resonance imaging and micro-computed tomography) and invasive techniques (histology and micro-dissection) to construct the annotated high-resolution three-dimensional dataset, or morphome, of the zebra finch (*Taeniopygia guttata*) syrinx. We identified and annotated syringeal cartilage, bone and musculature *in situ *in unprecedented detail. We provide interactive three-dimensional models that greatly improve the communication of complex morphological data and our understanding of syringeal function in general.

**Conclusions:**

Our results show that the syringeal skeleton is optimized for low weight driven by physiological constraints on song production. The present refinement of muscle organization and identity elucidates how apposed muscles actuate different syringeal elements. Our dataset allows for more precise predictions about muscle co-activation and synergies and has important implications for muscle activity and stimulation experiments. We also demonstrate how the syrinx can be stabilized during song to reduce mechanical noise and, as such, enhance repetitive execution of stereotypic motor patterns. In addition, we identify a cartilaginous structure suited to play a crucial role in the uncoupling of sound frequency and amplitude control, which permits a novel explanation of the evolutionary success of songbirds.

## Background

Birdsong has inspired lovers, musicians, poets, naturalists and scientists throughout our documented history. This fascination is fueled both by our appreciation of the natural beauty of these sound compositions and the intrinsic similarity with our own faculty of speech [1,2]. For birds, singing is of major importance to communicate with each other, helping them to establish and maintain territories and to signal reproductive fitness to mates [3-5]. The intricate physical and neural mechanisms by which the songs are perceived, produced and learned are an important substrate for sexual and natural selection, and song is therefore an important aspect driving speciation in birds [6,7].

More recently, songbirds have also developed into an important model system for answering some of the most fundamental questions in behavioral neuroscience [8]. In particular, songbirds have emerged as a widely used experimental animal model for understanding the neural mechanisms that underlie imitative vocal learning [9]. Like humans, songbirds are among the few animal groups that learn to produce species-specific vocalizations from a tutor [10]. This complicated behavior depends on the integrated action of neural systems for auditory perception, song learning, song production and processing of social information [8,9,11,12]. Many components of these specialized neural circuits have been identified and are referred to as the song system [8,9,13,14].

Songbirds produce sound using a uniquely avian vocal organ, the syrinx, which is located at the bifurcation of the trachea into the two primary bronchi of the lungs [15,16]. The syrinx was named by Huxley [17] to replace the confusing terminology of upper and lower larynx. For over 200 years, morphologists and systematists have used the cross-species diversity of syringeal anatomy to classify avian taxa. The syrinx of the more than 4,000 songbird (Aves: Passeriformes: Passeri) species exhibits substantial anatomical variation [18,19], but is morphologically conserved compared with other avian taxa [16,20]. Previous work has led to insights on syringeal biomechanics and song physiology, but experimental limitations have focused research primarily on larger songbird species [21-28]. In songbirds, sound is produced by air flow-induced oscillations of labia in each bronchus, which cause pressure fluctuations [24,29-33]. The fundamental frequency of sound and fast temporal dynamics of songs are predominantly controlled by the syrinx [28,34-37], and spectral composition is additionally affected by upper vocal tract filtering [38-40]. The fine-temporal structure of song is under the direct motor control of syringeal muscles [34,41-44]. The (sub)millisecond temporal precision present in telencephalic pre-motor areas [45-47] is actuated by superfast syringeal musculature [36], a rare muscle type found predominantly in association with sound production and modulation in vertebrates [48,49].

In the last decade, our understanding of the central processing of song production has advanced significantly [8,9,50] in the dominant avian model species for vocal production learning, the zebra finch (*Taeniopygia guttata *(Vieillot, 1817), Passeriformes: Estrildidae). Nevertheless, we currently lack crucial mechanistic insights into the function of these neural motor circuits and how their output is transformed into vocalizations by peripheral control and biomechanics of the complicated morphology of the syrinx [51,52]. This is particularly unfortunate because the song of the zebra finch and the development of its vocal imitation process is by far the best described bioacoustically [53-56], providing a rich substrate for peripheral structure-function studies. Elegant but extremely simplified models of sound production are capable of good qualitative fits of sound [29-31,33,57-59]. These models do not claim to capture the physical dynamics faithfully or include any functional morphology of the syrinx, but they have greatly enhanced our insights into how even driving forced oscillators by simple parameter changes can lead to complex patterns. This simplicity, however, limits these models' capacity to incorporate muscle function, and hence realistic neural control, stressing the need for computational approaches based on realistic geometries that may also explain the diverse syringeal morphologies from a biomechanical and evolutionary point of view.

We only have an incomplete understanding of how central motor areas connect to [11,60-63] and instruct [64] the peripheral musculature that controls sound production and how this musculature modulates the peripheral vocal organ to generate and modify sounds [51,52]. Even at the terminus of the vocal motor pathway, neural signals demonstrate relatively weak correlates with acoustic parameters [64], making the relationship between neural activity and sound production difficult to predict. Indeed, we still do not know how central song motor programs and the motor exploration of such programs are translated into sound.

To make the critical step that will allow for precise mapping of the song system onto the periphery, we first need to establish a detailed quantitative understanding of the structure and function of the zebra finch syrinx and its muscles. Only a more refined knowledge of syringeal geometry will permit obtaining an understanding of syringeal biomechanics and mechanical loads experienced *in vivo*. Further, neural control of sound parameters may be misinterpreted if a control parameter (for example, labial position) is affected by antagonistic activation of more than two muscles, in which case different combinations of recruitment levels can produce the same parameter value (for example, tension). If combinations of muscle activation, or synergies [65-67], control a parameter value, it is not individual muscle activation but the underlying synergy that functionally correlates with the control parameter. Therefore precise knowledge of muscle identity and attachments will allow for making better predictions about their antagonistic and synergistic effects.

Here we present an annotated high-resolution three-dimensional (3D) morphological dataset, which we have dubbed a morphome, that contains the geometry of the zebra finch syrinx in unprecedented detail. We used two non-invasive imaging techniques, high-field magnetic resonance imaging (MRI) and micro-computed tomography (μCT), in combination with invasive techniques, such as histology and micro-dissection, to annotate the dataset. We focus on structural elements affecting sound production, including the syringeal skeleton, soft tissues and cartilaginous parts, syringeal muscle definition and attachment. Our data provide the basis to study the micromechanics, exact innervation and neuromuscular control of the syrinx, and develop computational models based on realistic geometries. These efforts will be aided by interactive 3D models as a tool to improve understanding of syringeal morphology and function as well as the dissemination of complicated biological structures [68,69]. The data presented in this paper have been deposited on the songbird neuroscience community website http://www.songbirdscience.com.

## Results

**Figure 1:**
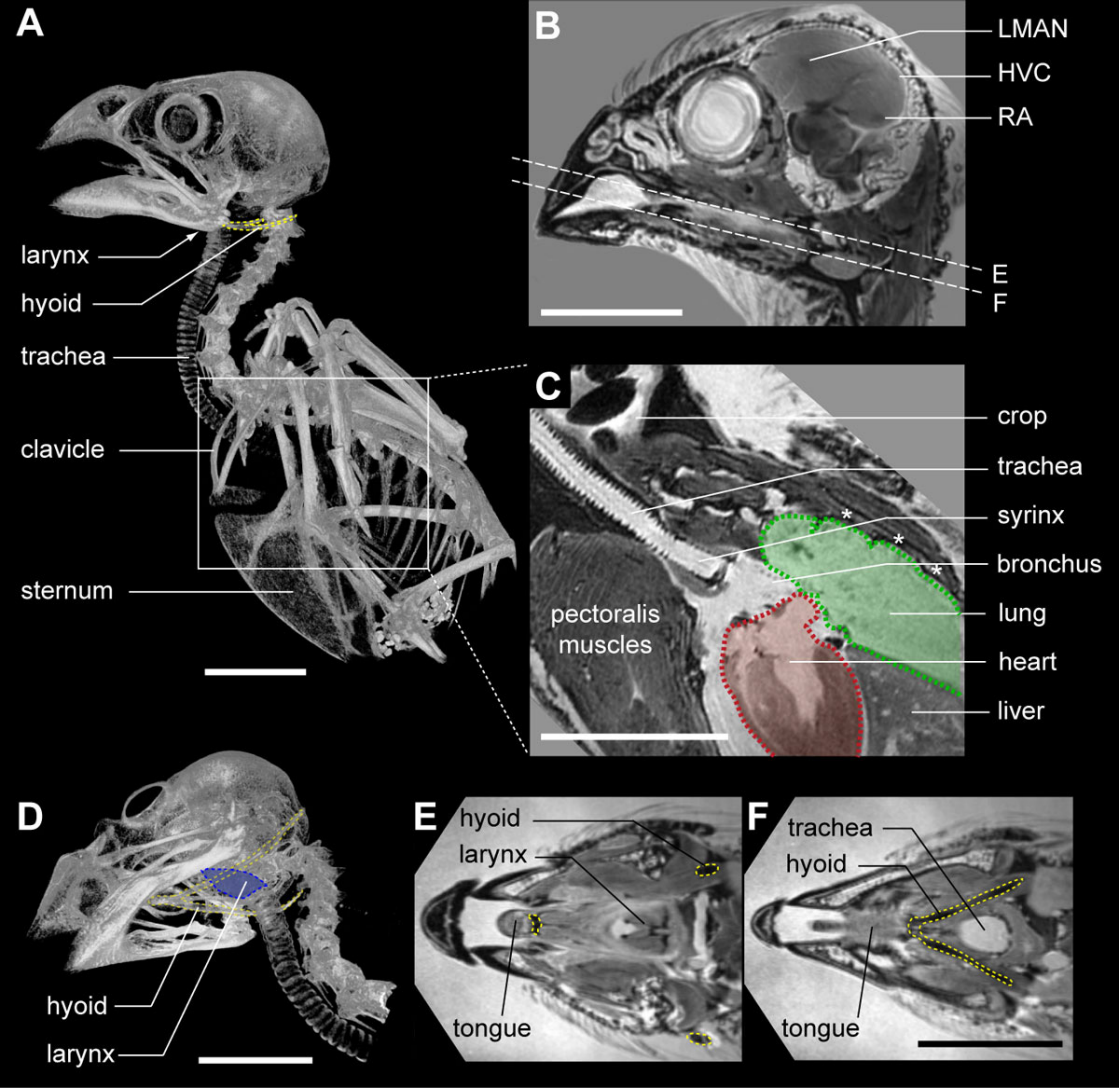
**Location of song system components in the male zebra finch**. **(A) **μCT-based volume rendering of the zebra finch skeleton. Boxed section shown enlarged in C. **(B) **Virtual sagittal section through a 3D high-field MRI dataset of the male zebra finch head. Visible are three nuclei of the song system: HVC (used as a proper name) and the robust nucleus of the arcopallium (RA) that both belong to the descending motor pathway, and the lateral portion of the magnocellular nucleus of the anterior nidopallium (LMAN), which belongs to the anterior forebrain pathway. Dotted lines indicate the virtual sections shown in E and F. **(C) **Virtual sagittal section through a 3D MRI dataset showing that the syrinx is located at the bifurcation of the trachea into the bilateral primary bronchi, close to the heart (red shaded outline) and lungs (green shaded outline). Note the dark seeds in the crop. The asterisks indicate intercostal muscles. **(D) **μCT-based volume rendering showing the hyoid bone (yellow) and larynx (blue), which are upper vocal tract modulators important in the filtering of sound properties [38,40]. **(E,F) **Virtual transversal sections through a 3D MRI dataset of the head as indicated in (B) showing the tongue, larynx, trachea (blue) and hyoid (yellow dotted line). Scale bars: 10 mm.

The brain nuclei that comprise the song system in the zebra finch (Figure 1A,B) contain motoneurons that connect with three major muscle systems: the respiratory system, including air sacs, modulated by intercostal and abdominal muscles; the syringeal musculature; and the suprasyringeal vocal tract, including trachea, larynx, oropharyngeal space and beak, modulated mainly by laryngeal, hyoid and orofacial muscles (Figure 1C-F). The zebra finch syrinx is located at the bifurcation of the trachea into the two primary bronchi, just anterior to the lungs and heart (Figure 1C). We constructed a 3D dataset of the syrinx with a 5-μm isotropic voxel resolution based on high-resolution μCT scans. We created a morphome of the syrinx by annotating structures in this dataset based on the μCT scans with complementary guidance by high-field MRI, conventional histology and micro-dissection (see Methods). We will present this dataset starting with the ossified skeleton of the syrinx, subsequently adding on the other tissues, such as cartilage, sound-producing labia and finally muscles. We will then summarize our results, focusing on the motor actuation of syringeal elements and syringeal stability *in situ*. Table 1 provides an overview of the nomenclature and abbreviations used in text and figures. We include two interactive 3D models (Additional files 1 and 2) for which we have included several predefined views that correspond to our two-dimensional (2D) figures below.

**Table 1:** Overview and abbreviations of anatomical structures associated with the zebra finch syrinx.

English term	Latin term^a^	Brief anatomical description	Figure(s)
bronchial half-ring (B1, B2, B3)	*cartilago bronchosyringealis *(App. Resp. 42)	Paired, ossified, irregularly-shaped cartilage. Located at the rostral end of the primary bronchus. Modified bronchial cartilage.	2, Additional file 1, 4
bronchial half-ring (B4, B5...)	*cartilago bronchialis *(App. Resp. 52)	Paired, ossified, C-shaped cartilage. Forms the lateral, ventral and dorsal walls of the primary bronchus.	2, Additional file 1, 4
clavicular air sac (CAS)	*saccus clavicularis *(App. Resp. 81)	Unpaired, air-filled sac surrounding the syrinx. Located in-between the clavicles.	12
clavicular air sac membrane (CASM)	*membrana sacci clavicularii *(present study)	Membrane surrounding clavicular air sac and interbronchial foramen. Provides attachment for various muscles.	8, 9, 12
deep dorsal syringeal muscle (DDS)	*musculus syringealis dorsalis profundus *(present study)	Paired muscle. Attaches to tympanum and to bronchial half-ring B2.	Additional file 2, 8
deep ventral tracheobronchial muscle (DVTB)	*musculus tracheobronchialis ventralis profundus *(present study)	Paired muscle. Attaches to tympanum and to bronchial half-ring B3.	Additional file 2, 8
dorsal syringeal muscle	*musculus syringealis dorsalis *(App. Resp. 51)	This muscle is divided into three parts: the medial dorsal syringeal muscle, the lateral dorsal syringeal muscle and the deep dorsal syringeal muscle.	Additional file 2, 8
dorsal tracheobronchial muscle (DTB)	*musculus tracheobronchialis dorsalis *(App. Resp. 51)	Paired muscle. Attaches to clavicular air sac membrane and to bronchial half-ring B2.	Additional file 2, 8
interbronchial foramen (IBF)	*foramen interbronchiale *(App. Resp. 46)	Unpaired, air-filled sac. Located between the bifurcation of the trachea and the interbronchial ligament. A diverticulum of the clavicular air sac.	6, 9
interbronchial ligament (IBL)	*ligamentum interbronchiale *(App. Resp. 46)	Unpaired connective tissue element. Located caudal to the bifurcation of the trachea as well as the interbronchial foramen. Connects left and right primary bronchus.	7 - 9
lateral dorsal cartilage (LDC)	*cartilago dorsalis lateralis *(present study)	Paired cartilaginous pad. Embedded dorsally within the medial tympaniform membrane.	7, Additional file 2
lateral dorsal syringeal muscle (LDS)	*musculus syringealis dorsalis lateralis *(present study)	Paired muscle. Attaches to tracheal rings T3 and T4 as well as to bronchial half-ring B1.	Additional file 2, 8
lateral labium (LL)	*labium laterale *(App. Resp. 49)	Paired connective tissue element. Projects from the medial part of bronchial half-ring B3 into the airway of the syrinx.	7, Additional file 2
lateral ventral cartilage (LVC)	*cartilago ventralis lateralis *(present study)	Paired cartilaginous pad. Ventrally continuous with bronchial half-ring B3. Provides attachment for the superficial ventral tracheobronchial muscle.	7, Additional file 2
medial dorsal cartilage (MDC)	*cartilago dorsalis medialis *(present study)	Paired cartilaginous pad. Embedded dorsally within the medial tympaniform membrane. Provides attachment for the medial dorsal syringeal muscle.	7, Additional file 2
medial dorsal syringeal muscle (MDS)	*musculus syringealis dorsalis medialis *(present study)	Paired muscle. Attaches to tracheal rings T3 and T4 as well as to the medial dorsal cartilage.	Additional file 2, 8
medial labium (ML)	*labium mediale *(App. Resp. 49)	Paired connective tissue element. Projects from the lateral wall of the pessulus into the airway of the syrinx. Forms a tissue continuum with the medial tympaniform membrane.	7, Additional file 2
medial tympaniform membrane (MTM)	*membrana tympaniformis medialis *(App. Resp. 48)	Paired connective tissue element. Suspended between the free ends of bronchial half-rings B1, B2 and B3. Forms a tissue continuum with the medial labium.	7
medial ventral cartilage (MVC)	*cartilago ventralis medialis *(present study)	Paired cartilaginous pad. Ventrally continuous with bronchial half-ring B2. Embedded ventrally within the medial tympaniform membrane. Provides attachment for the ventral syringeal muscle.	7, Additional file 2, 10
medial vibratory mass (MVM)	*corpus vibratorius medialis *(present study)	Paired tissue continuum consisting of medial labium and medial tympaniform membrane.	7
pessulus (PES)	*pessulus *(App. Resp. 43)	Unpaired, ossified cartilage. Located at the caudal end of the tympanum. Derived from the fusion of two bronchial half-rings.	2, 3
primary bronchus (PBR)	*bronchus primarius *(App. Resp. 52)	Paired tube composed of soft and hard tissue. Connects the trachea with the secondary bronchus that is located within the lungs.	6
short tracheobronchial muscle (STB)	*musculus tracheobronchialis brevis *(App. Resp. 51)	Paired muscle. Attaches to tympanum and to bronchial half-ring B2.	Additional file 2, 8
sternotracheal muscle (ST)	*musculus sternotrachealis *(App. Resp. 50)	Paired muscle. Attaches to tracheal ring T1 and to a protrusion of the sternum close to the coracoid.	Additional file 2, 8
superficial ventral tracheobronchial muscle (SVTB)	*musculus tracheobronchialis ventralis superficialis *(present study)	Paired muscle. Attaches to tracheal rings T4 and T5 as well as to bronchial half-ring B3.	Additional file 2, 8
trachea (TRA)	*trachea *(App. Resp. 36)	Unpaired tube composed of numerous tracheal rings connecting the larynx to the lungs.	1, 2, 6, 10
tracheal ring (T1, T2...)	*cartilago trachealis *(App. Resp. 36)	Unpaired, ossified cartilage. The trachea is composed of numerous tracheal rings.	2, 3
tracheolateral muscle (TL)	*musculus tracheolateralis *(App. Resp. 50)	Paired muscle. Forms an extended band along the trachea. Attaches caudally to the syrinx and rostrally to the larynx.	9
tympanum (TYM)	*tympanum *(App. Resp. 39)	Unpaired, ossified cylinder. Located at the caudal end of the trachea. Formed by the close apposition or fusion of four to six tracheal rings and one paired bronchial half-ring.	2, 3
ventral syringeal muscle (VS)	*musculus syringealis ventralis *(App. Resp. 51)	Paired muscle. Attaches to tympanum and to the medial ventral cartilage.	Additional file 2, 8
ventral tracheobronchial muscle (not used here)	*musculus tracheobronchialis ventralis *(App. Resp. 51)	This muscle is divided into two parts: the superficial ventral tracheobronchial muscle and the deep ventral tracheobronchial muscle.	-

Structural components are presented in alphabetical order. The abbreviations of the English terms provided here are used throughout the manuscript. ^a^App. Resp. refers to the chapter *Apparatus Respiratorius *by King [72] in Baumel and colleagues [130], while the numbers indicate the respective section of that chapter.

### Ossified elements of the zebra finch syrinx

**Figure 2:**
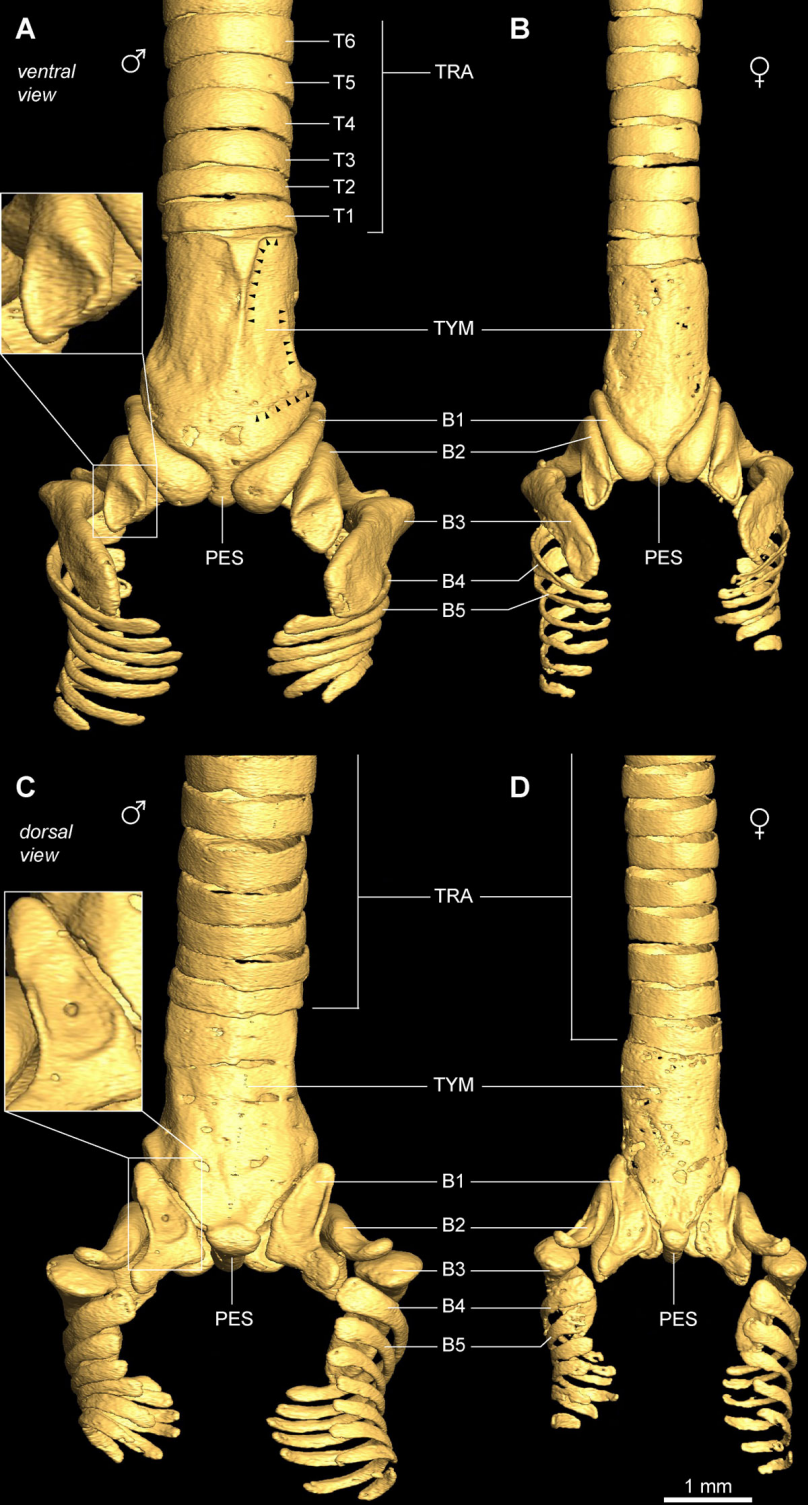
**Ossified structural elements of the male and female zebra finch syrinx**. **(A) **Ventral and **(C) **dorsal view of male syringeal skeleton surface rendering based on μCT datasets with 5 μm isotropic voxel resolution. **(B) **Ventral and **(D) **dorsal view of the female syringeal skeleton. Muscle attachment sites leave impressions on the surface of the tympanum (black arrowheads in A) and on bronchial half-rings B1 and B2 (insets). Abbreviations as listed in Table 1.

**Figure 3:**
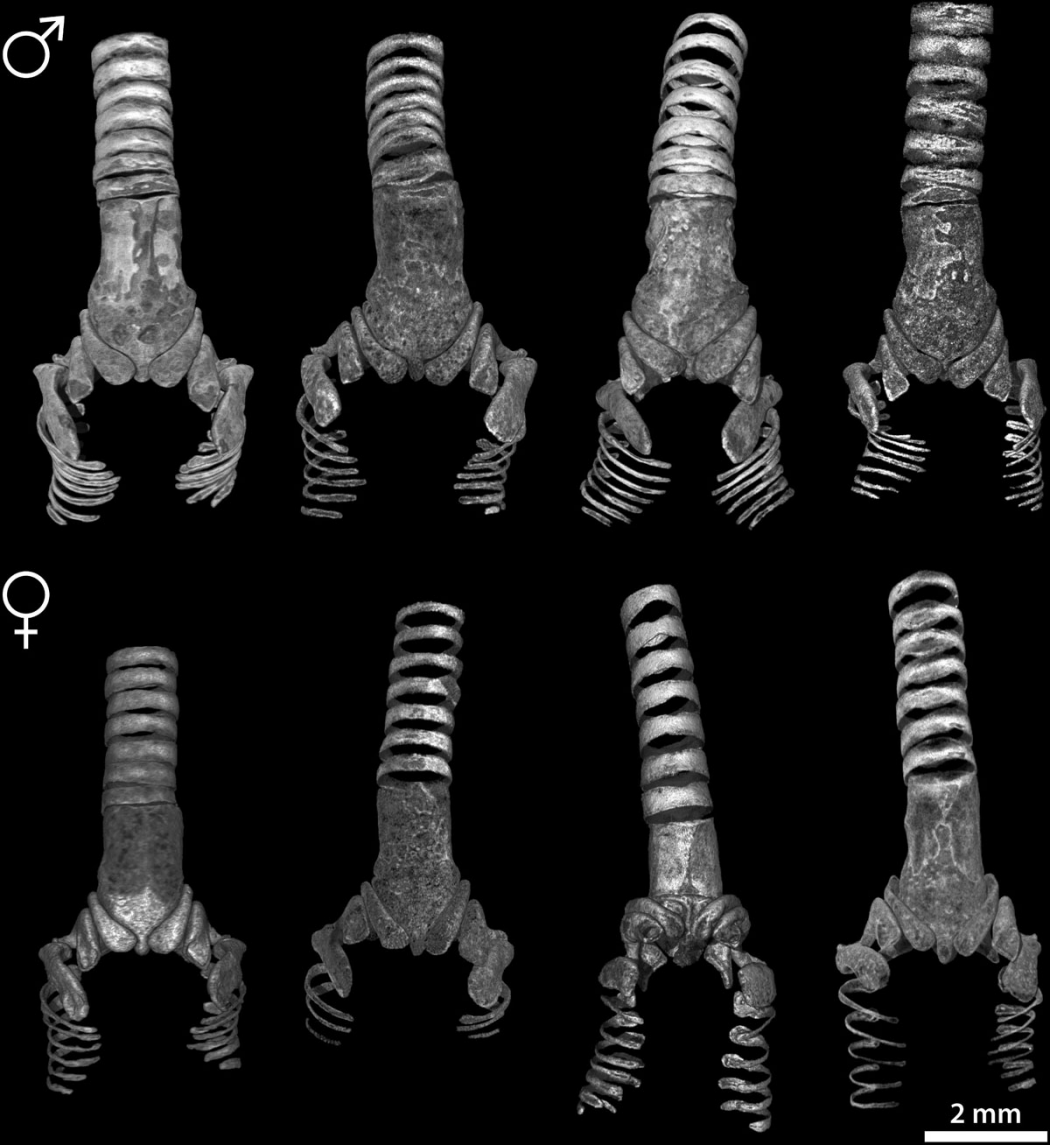
**Selection of male and female syringeal skeletons based on volume rendered μCT datasets**.

We visualized and compared the ossified elements of the male (N = 12) and female (N = 5) zebra finch syrinx μCT datasets. Without staining of soft tissues, only ossified tissue is clearly visible in μCT scans. Figure 2 shows the 2D projections of a male and female 3D syringeal skeleton. Because no apparent asymmetries were observed in the number of bones comprising the syringeal skeleton (N = 17), we consider the structure symmetrical in our description. The zebra finch syringeal skeleton is clearly sexually dimorphic, with the male syrinx being larger and more robust (Figures 2 and 3, and Additional file 1). We observed considerable larger variation in the shape of the ossified elements in the female skeleton compared with the male skeleton (Figure 3). However, we found no differences in number and orientation of ossified elements between sexes (Figure 3). Therefore, we assume the following description to be accurate for both sexes.

**Figure 4:**
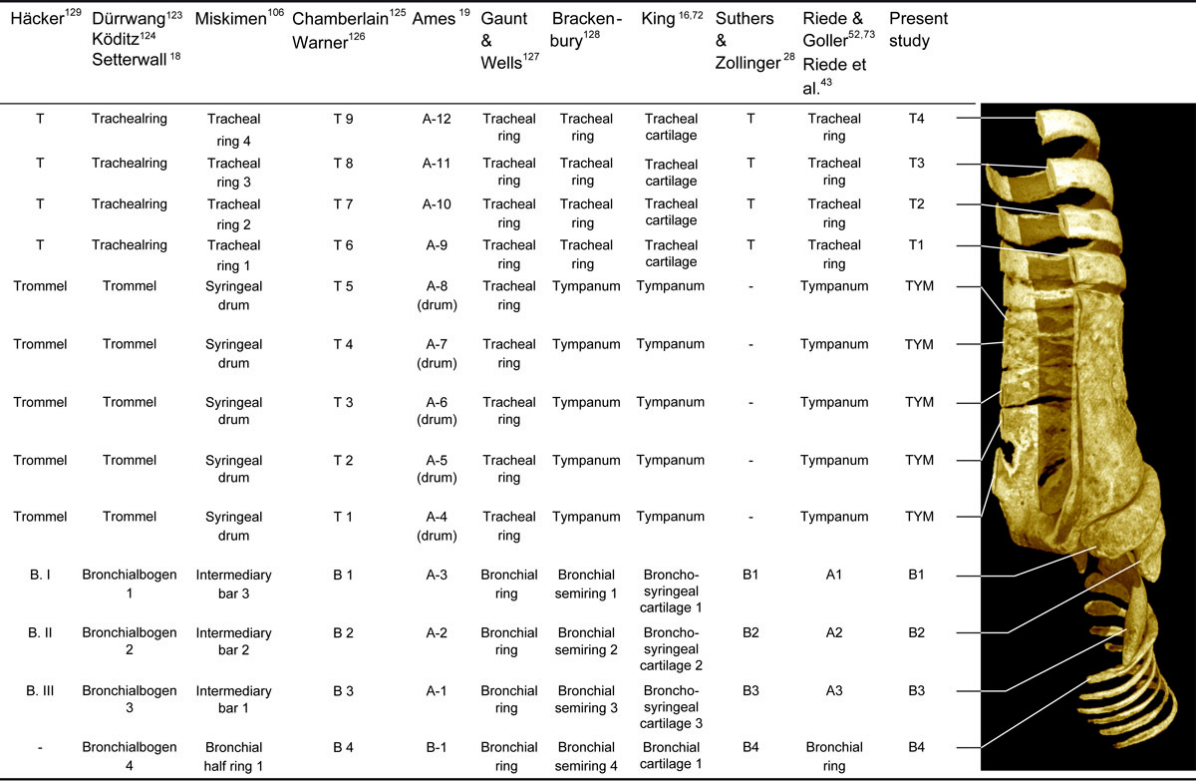
**Overview of terminology describing ossified structural elements of the songbird syrinx**. Alternative nomenclatures of previous authors [16,18,19,28,43,52,72,73,106,123-129]. The system that we adopt in this study is a combination of terminologies proposed by Häcker [129], Chamberlain and colleagues [125], and Warner [126]. Note in particular the approach proposed by Ames [19], which emphasizes the microstructure of individual syringeal bones that make up the tympanum. Abbreviations as listed in Table 1.

The zebra finch syringeal skeleton consists of modified tracheal rings and bronchial half-rings. It is therefore considered a syrinx of the tracheobronchial type [16]. Two bronchial half-rings and four to six tracheal rings are fused to form a rigid cylinder named the tympanum (Figure 2 and Additional file 1). At the tympanum's caudal end, two bronchial half-rings form a medial dorso-ventral bridge called the pessulus (Figure 2 and Ca*udal view *in Additional file 1). The trachea consists of tracheal rings (T1, T2, and so on) rostral to the tympanum. Caudal to the tympanum are bronchial half-rings (B1, B2, and so on), of which the first three (B1 to B3) are highly modified (Figure 2). Despite the above-mentioned observation that the most caudal part of the tympanum consists of two bronchial half-rings, we decided not to rename them B1 to avoid excessive alterations to existing nomenclature. Figure 4 lists our nomenclature together with the alternative nomenclature of previous authors.

The syringeal muscles that attach directly onto bone surface leave clearly visible imprints. This is especially evident on the ventral side of the tympanum and also on the dorsal side of rings B1 to B3, where deep concavities indicate muscle attachment sites (Figure 2 and Additional file 1). These impressions are apparent, because bone is a dynamic tissue that is remodeled continuously as a result of local compressive and tensile stress [70]. Consequently, local forces exerted by muscles shape the surface microstructure of skeletal elements.

**Figure 5:**
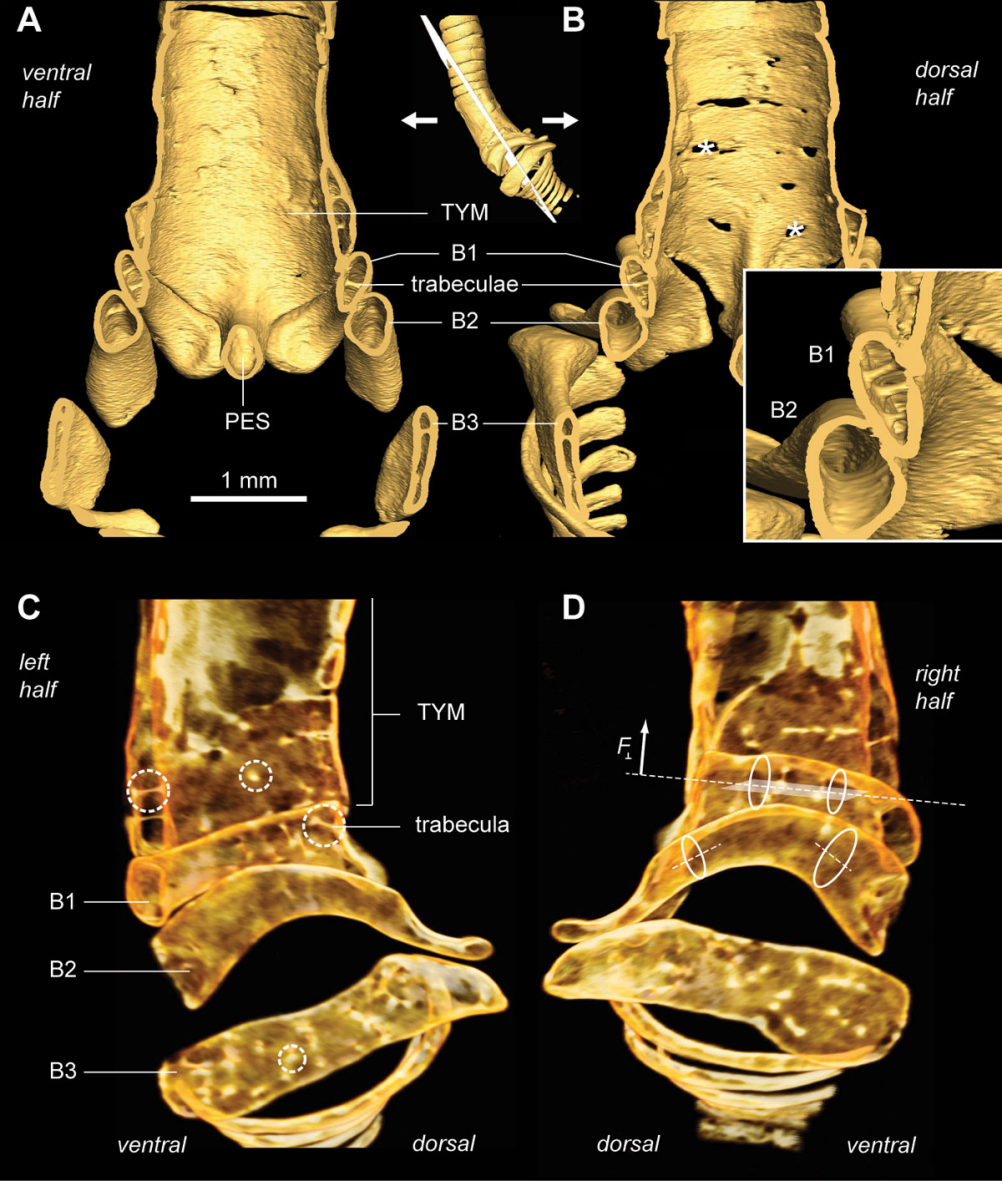
**The internal bone structure of the male zebra finch demonstrates optimization by combining low weight with strength**. **(A) **Ventral and **(B) **dorsal halves of a clipped bone surface rendering of non-contrasted μCT scan of the male syrinx, revealing the inside surface of the syrinx and cross-sectional views of the bronchial rings. The bronchial half-rings are hollow, laterally flattened, thin-walled bones fortified with trabeculae. The holes (asterisks) in the tympanum indicate lower X-ray attenuation values due to very thin walls or lower-density bone. The boxed inset shows a detailed view of bronchial half-rings B1 and B2 with trabeculae in bronchial half-ring B1. **(C) **Medial view of a semi-transparent volume rendering of the left hemisyrinx. Trabeculae can be seen as bright bars or dots, when seen on-axis, due to high density bone tissue (dashed circles). **(D) **Medial view of right hemisyrinx. Lateral flattening (ellipses) of bronchial half-rings increases their resistance to bending in the horizontal plane (dotted line and shaded plane) and therefore increases the maximal perpendicular force ($F_{⊥}$) that can be applied before mechanical failure occurs due to breaking [75,76]. The trabeculae prevent failure of the bones due to buckling [76]. Abbreviations as listed in Table 1.

Bronchial half-ring B1 is the first of the three heavily modified bronchial half-rings, whose geometry can be best understood by rotating the 3D models in Additional file 1. Bronchial half-ring B1 is hollow and arc-shaped seen from a lateral view and flattened in transverse section (Figure 5A,B). It fits tightly to the tympanum and its internal walls form a smooth surface (Figure 5A). In addition, several trabeculae structurally connect the inner and outer walls of B1 (Figure 5). The dorsal end of B1 contains no trabeculae (Figure 5C,D), is rostro-caudally flattened, and has a concave muscle attachment site (Figure 2C and Additional file 1). The dorsal apex of B1 has a lateral protuberance of which the caudal side forms a smooth arc with the pessulus and B4 when seen from a dorsal viewpoint (Additional file 1, *Cut dorsolateral view with labels*). The second bronchial half-ring (B2) is also arc-shaped, hollow and slightly flattened. Both the ventral and dorsal side have cup-shaped muscle attachment sites (Figure 2). In contrast with B1, B2 has no internal trabeculae (Figure 5). The third bronchial half-ring (B3) is the least arced of the three half-rings but also hollow, laterally flattened and heavily reinforced with trabeculae. The dorsal end widens to about four times its medial width and is characterized by a domed surface on the rostral end, which forms a joint with the flattened dorsal cup of B2 (Additional file 1, *Rostral view*).

The geometry and internal structure of the ossified skeleton determine how external forces are conducted by, through and in-between bones forming linkages and articulation points. As such, the skeleton provides a basis for the understanding of syringeal biomechanics. Several muscle attachment areas are clearly delineated by depressions and ridges on the syringeal skeleton.

### Non-muscular soft tissue elements of the zebra finch syrinx

**Figure 6:**
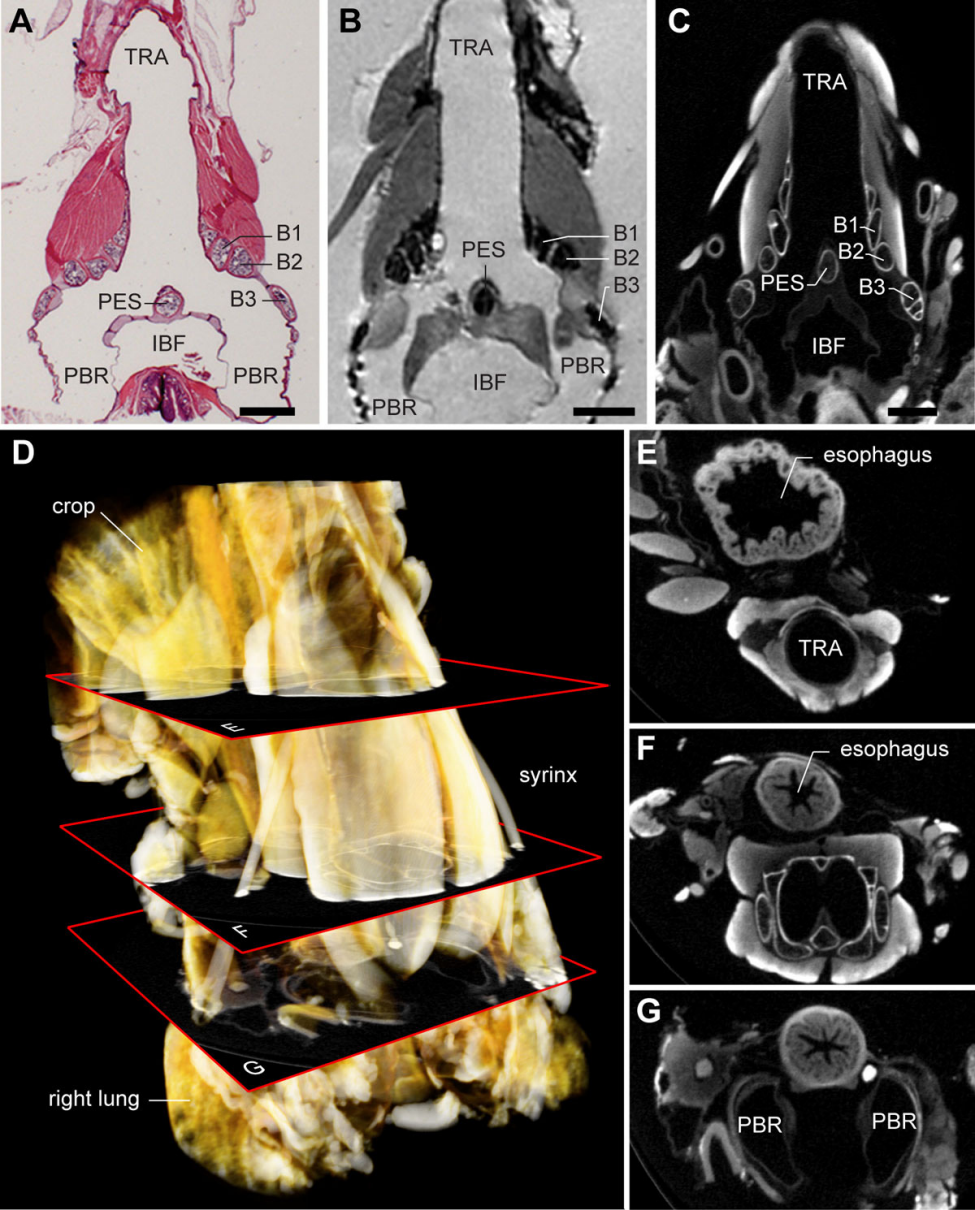
**Direct comparison of syringeal soft tissue imaging techniques reveals advantages of μCT**. **(A) **Frontal section of a male zebra finch syrinx prepared with conventional histology and stained with hematoxylin and eosin. Muscles appear as bright red, cartilage as speckled blue/purple. **(B) **Virtual frontal section through a 3D MRI dataset of a Magnevist-contrasted male zebra finch syrinx with isotropic voxel resolution of 23 μm. **(C) **Virtual frontal section through a 3D μCT dataset of an iodine-contrasted male zebra finch syrinx with an isotropic voxel resolution of 5 μm. The non-destructiveness, high resolution and relatively short scanning times of contrasted samples made μCT the optimal technique for the construction of the syrinx morphome. **(D) **Volume rendering of an iodine-contrasted μCT scan. **(E-G) **Virtual horizontal sections through the 3D μCT dataset at different positions as shown in D. Special effort was made to fix and scan the syrinx *in situ*, with surrounding tissues remaining intact. Abbreviations as listed in Table 1. Scale bars: 1 mm.

To image soft tissues in 3D at high resolution, we compared results derived from three methods: high-field (17.6 T) MRI with added contrast agent; μCT with two different types of staining; and conventional histology. Figure 6A-C shows a histological section next to two virtual sections through datasets based on MRI and μCT. With classical histology it is rather problematic to construct accurate 3D datasets, because of section alignment inaccuracies as well as unavoidable tissue distortions resulting from fixation, embedding, cutting and staining. The μCT technique provided the highest resolution (5 μm) datasets and was therefore used to generate 3D datasets for further study and annotation. Additional iodine-based staining (see Methods) provided sufficient contrast for unambiguous distinction of all syringeal soft tissues (Figure 6C). We took special care to scan the excised syrinx *in situ *by leaving all surrounding tissues, such as the lungs, esophagus, crop and large blood vessels, attached and intact (Figure 6D-G).

**Figure 7:**
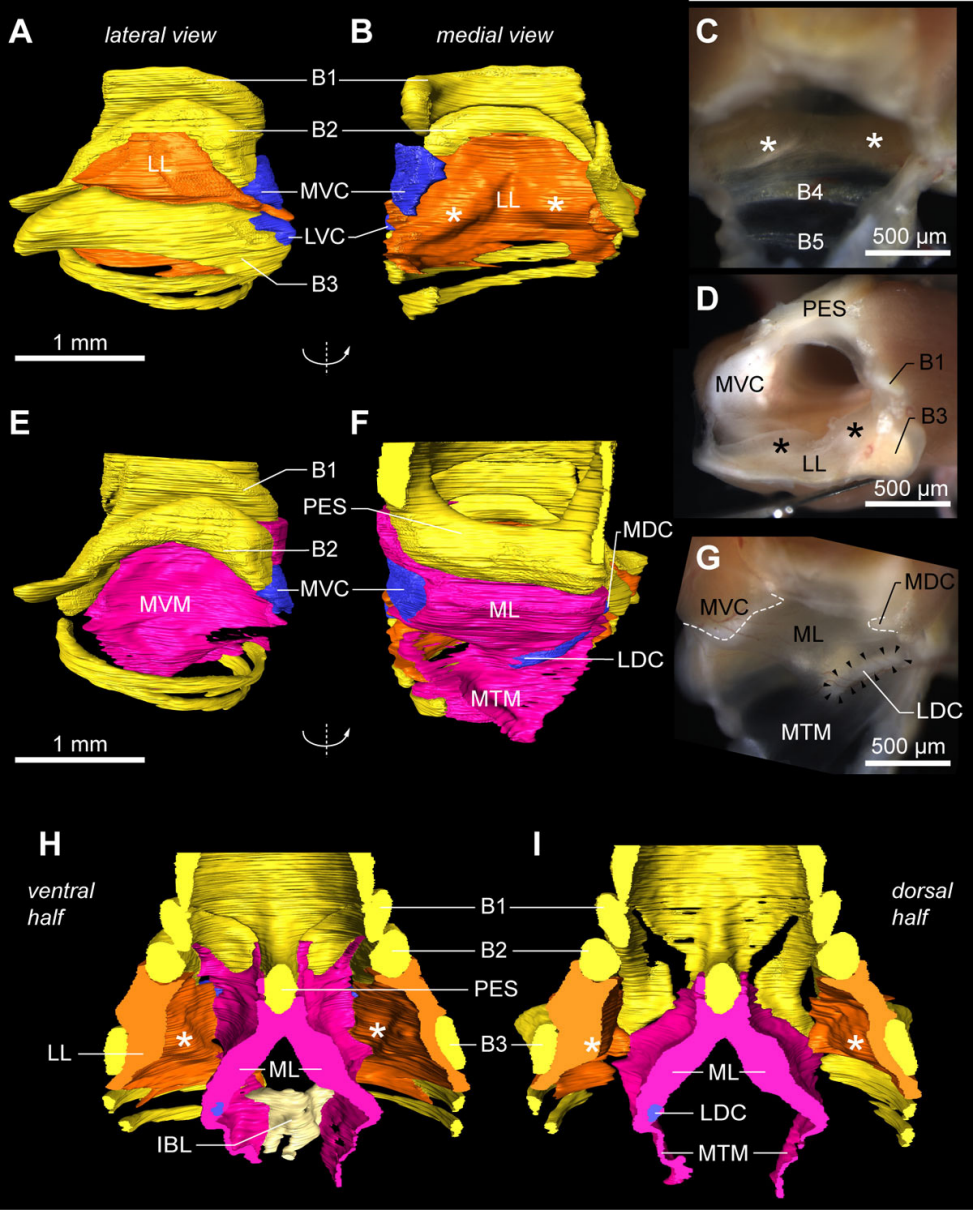
**The vibratory soft tissues in the primary bronchi**. **(A) **Lateral view onto the lateral labium (LL, orange) of the male right hemisyrinx. Cartilaginous tissue (blue) extends from bronchial half-rings B2 and B3. The MVC (blue), a large cartilaginous pad, extends from B2 and bends medially. **(B) **Same structure as in A, but turned 180° around the vertical axis, providing a medial to lateral view onto the LL. The LL is connected to the medial side of bronchial half-ring B3 and is thickened in two bands (asterisks). **(C) **Same medial view as in B onto right LL of freshly dissected male right hemisyrinx (MVM removed). **(D) **Caudal view onto left LL of left hemisyrinx with left primary bronchus removed. **(E) **Lateral view on the medial vibratory mass (MVM, pink), the tissue continuum comprising ML and medial tympaniform membrane (MTM). Bronchial half-ring B3 is removed for an unobscured view onto the MVM, which attaches to the pessulus (PES) and forms the medial wall of the primary bronchi. **(F) **Same structure as in E, but turned 180° giving a medial to lateral view. The MVC is embedded in the ventral part of the MVM, as well as two small other cartilaginous pads: the medial dorsal cartilage (MDC) and the lateral dorsal cartilage (LDC). **(G) **Same view as in F of a freshly dissected right hemisyrinx with the musculature left intact. **(H) **Surface renderings of the ventral and **(I) **dorsal half of the internal bone structure of the syrinx with sound-producing labia. Abbreviations as listed in Table 1.

The bilateral sound-producing vibrating tissues in the songbird syrinx [24,26,30,32] are located in the primary bronchi (Figure 7). Additional file 2 contains the interactive 3D model in which all structures are switched on initially. Although both left and right sides can be interactively accessed in Additional file 2, we will here focus on the right side of the male syrinx because it is predominantly used during zebra finch vocalization [71]. The lateral labium is connected to the medial side of half-ring B3 from dorsal to ventral (Figure 7A,B and Additional file 2, view *Lateral labium*) and thickens in two rostro-caudal running bands (Figure 7B), as also observed in all our fresh tissue micro-dissections (Figure 7C,D). On its medial side, each bronchus is sealed airtight from the pessulus to the lungs by the medial vibratory mass (Figure 7E and Additional file 2, view *Medial vibratory mass*). This tissue continuum [32] is roughly triangular-shaped from a medio-lateral view and consists of a thicker rostral part, the medial labium that connects dorso-ventrally to the pessulus, and a thinner caudal part, the medial tympaniform membrane (Figure 7F). We found no evidence of a *membrana semilunaris *on top of the pessulus in the zebra finch syrinx.

Dissections and μCT scans reveal the presence of four paired structures consisting of cartilaginous tissue (Figure 7 and Additional file 2). First, bronchial half-ring B2 has a large medial ventral cartilaginous extension in both males and females, which shows some ossification at the ends in some individuals. This structure connects to the ventral side of the ML. Setterwall [18] named this structure the *cartilago tensor*, but we here adopt a terminology modified after King [72] by naming this cartilaginous pad the medial ventral cartilage (MVC). Second, more lateral, a cartilaginous rostro-ventral extension of B3 can be observed, the lateral ventral cartilage. In addition, two cartilaginous elements are embedded in the medial labia on the dorsal side. Third, a disc-shaped dorsal element, the medial dorsal cartilage, is situated in the medial labia just caudal of the pessulus. Fourth, an elongated element, the lateral dorsal cartilage, is located on the border between the medial labia and the medial tympaniform membrane. This latter structure was named *cartilago tensor *in [32].

Summarizing, we provide, to our knowledge, a first 3D description of the sound-producing vibratory tissues including their embedded cartilaginous linkages *in situ *using the complementary approaches of conventional histology, micro-CT and MRI.

### Musculature of the zebra finch syrinx

**Figure 8:**
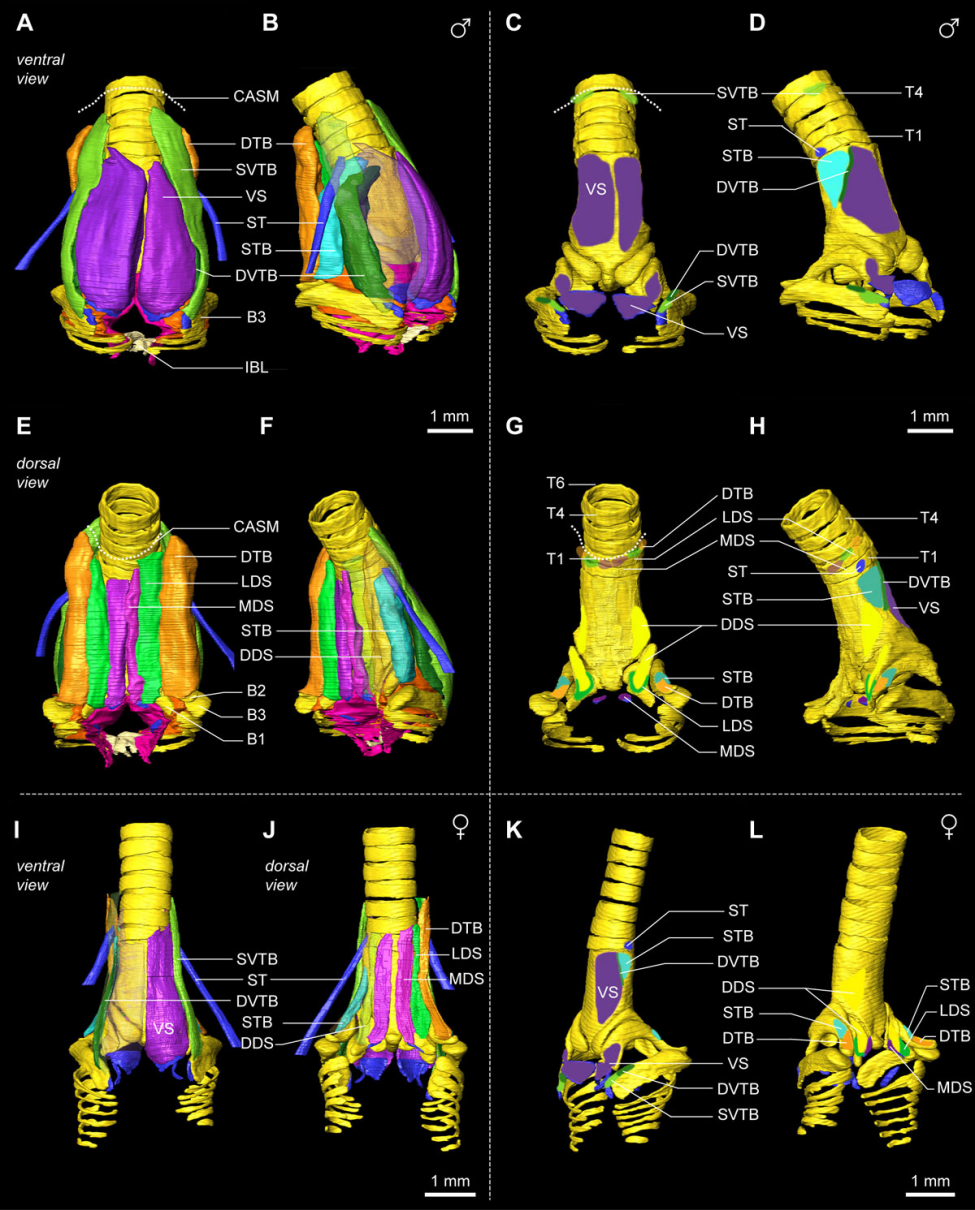
**Syringeal muscles and their attachment sites in the male and female zebra finch syrinx morphome**. **(A) **Ventral view of the male zebra finch syrinx morphome based on an iodine-stained μCT scan showing muscles, bones, cartilaginous pads, and sound-producing labia. **(B) **Ventro-lateral view with transparent right ventral muscles VS and SVTB, revealing the underlying DVTB. **(C) **Attachment sites of the syringeal muscles seen from ventral and **(D) **ventro-lateral. The dotted line indicates the location of the CASM on which several muscles insert. **(E) **Dorsal view of the male zebra finch syrinx morphome. **(F) **Ventro-lateral view with transparent right dorsal muscles SVTB and LDS, revealing the DDS and DVTB. **(G) **Attachment sites of the syringeal muscles seen from dorsal and **(H) **dorso-lateral. **(I) **Ventral view of the female zebra finch syrinx morphome showing muscles, bones, cartilaginous pads and sound-producing labia. The muscles VS, SVTB and DVTB on the right hemisyrinx are transparent. **(J) **Dorsal view with left MDS and DTB transparent. **(K) **Muscle attachment sites seen from ventro-lateral and **(L) **dorso-lateral view. Abbreviations as listed in Table 1. DDS: yellow; DTB: orange; DVTB: dark green; LDS: neon green; MDS: violet; ST: blue; STB: cyan; SVTB: light green; VS: purple.

Syringeal muscles translate the motor commands from the central song system into syringeal behavior. Therefore it is critical to identify and locate all syringeal muscles. We identified the muscles controlling the zebra finch syrinx and established their orientation and attachments sites using a combination of micro-dissection and μCT scan annotation (see Methods). Vertebrate skeletal striated muscle tissue is hierarchically organized. A muscle fiber is a single multinucleated cell containing contractile elements, a fiber bundle is a collection of muscle fibers, and numerous fiber bundles make up a muscle. In contrast to most other skeletal muscles, we observed that syringeal muscle fiber bundles are not organized in cylindrical bundles, but instead in well-defined parallel sheets containing dozens to hundreds of muscle fibers. We will first focus on the muscles that have both attachment sites on the syrinx, the intrinsic syringeal muscles [16], and subsequently include the muscles with one attachment site outside of the syrinx, the extrinsic syringeal muscles. Figure 8 shows the syringeal muscles and their attachment sites for the male and female zebra finch syrinx. Additional file 2 contains the 3D model of the male syrinx morphome with all annotated structures and predefined views as seen in Figure 8. Although sexual dimorphism in muscle volume is obvious, we found no differences in muscle definition and attachment sites between male and female specimens. Therefore the description below applies to both sexes.

**Figure 9:**
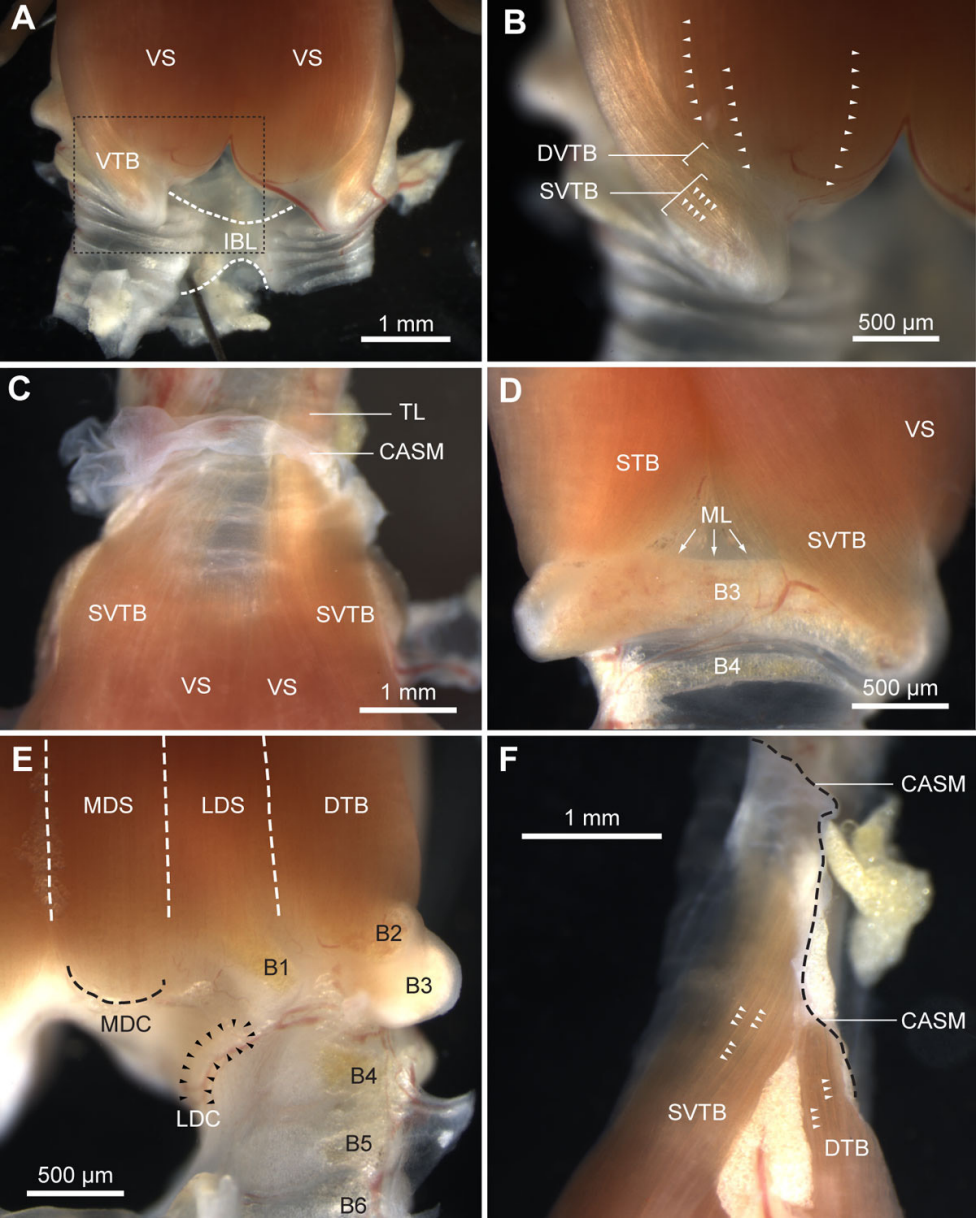
**Syringeal muscles of a freshly dissected male zebra finch syrinx**. **(A) **Ventral view of the caudal part of the syrinx showing the ventral muscles VS and VTB, and the IBL. The IBL connects the primary bronchi and restricts their lateral movement. Dashed box is shown enlarged in B. **(B) **Detail of VTB attachment on bronchial half-ring B3. Syringeal muscles are organized in sheets of muscle fibers (arrowheads). **(C) **Close-up of the rostral attachment sites of the ventral muscles. The VS fibers end on the edge of the tympanum, while the SVTB fibers do not attach onto bone but end in the connective tissue of the CASM. Rostral to the CASM, the extrinsic TL attaches and runs along the trachea up to the larynx. **(D) **Lateral view of the right hemisyrinx showing the muscle attaching on B3. Some fibers of the STB attach directly on the ML that is located on the inside of B3. **(E) **Dorsal muscle attachment sites. **(F) **Lateral view of rostral attachment sites of SVTB and DTB clearly reveals the organization of syringeal muscles in sheets (arrowheads). Individual muscle fibers can be seen within the fiber bundle sheets. The edge of the CASM is indicated by the dashed black line. Abbreviations as listed in Table 1.

We identified two muscles on the ventral side of the syrinx: *musculus syringealis ventralis *or ventral syringeal muscle (VS) and *musculus tracheobronchialis ventralis *or ventral tracheobronchial muscle (VTB), of which the latter consists of two parts: *musculus tracheobronchialis ventralis profundus *or deep ventral tracheobronchial muscle (DVTB) and *musculus tracheobronchialis ventralis superficialis *or superficial ventral tracheobronchial muscle (SVTB) (Figure 8A,B). The VS is the largest syringeal muscle and its caudal attachment site is located in the cup of bronchial half-ring B2 and on the MVC, continuous with the ventral end of B2 (Figures 8C,D and 9A). Rostrally, the attachment site, which can be clearly seen as an impression in Figure 2 and Additional file 1 starts on the mid part of the tympanum and extends to the top of the tympanum (Figure 8C,K). The VS is organized as a series of muscle sheets that run parallel to the central axis of the syrinx (Figure 9B). The VTB has previously been considered as a single muscle [73], but using our μCT data supported by micro-dissection, it became apparent that the VTB is made up of two parts with distinct rostral attachment sites. The caudal attachment site for SVTB fibers is located on the rostro-ventral tip of B3 (Figures 8C,D and 9B). The rostral attachment site is not on bone but instead on the clavicular air sac membrane (CASM) at tracheal rings T4 and T5 (Figures 8C,D and 9C). The caudal attachment site for the DVTB is located on B3 more proximal to the attachment site of the SVTB (Figures 8CD and 9B). Both the SVTB and the DVTB are externally apparent at this point, but, more rostrally, the DVTB twists underneath the SVTB and is no longer externally visible. The rostral attachment site of the DVTB is a very thin lateral strip on the tympanum, running parallel to the central axis of the syrinx (Figure 8D,K).

The dorso-lateral musculature consists of three intrinsic syringeal muscles that consist of five different parts. Although these muscles run in close proximity to each other, they attach to separate syringeal bones and therefore actuate the syringeal skeleton differently. First, the *musculus syringealis dorsalis *or dorsal syringeal muscle consists of three parts: *musculus syringealis dorsalis medialis *or medial dorsal syringeal muscle (MDS), *musculus syringealis dorsalis lateralis *or lateral dorsal syringeal muscle (LDS) and *musculus syringealis dorsalis profundus *or deep dorsal syringeal muscle (DDS). The most medially located muscle, the MDS, attaches caudally on the medial dorsal cartilage that is embedded in the dorsal part of the ML (Figures 8G and 9E). Rostrally, the MDS ends in connective tissue, continuous with the CASM, and not on the tympanum directly. The LDS attaches caudally onto the ridge around the cup on the ventral end of bronchial half-ring B1. Both MDS and LDS run parallel to the central axis of the syrinx and are organized in a series of muscle sheets (Figure 9E). Their rostral attachment sites are not on the tympanum, but on the CASM near tracheal ring T2. The DDS is the only syringeal muscle that is not visible externally. The caudal attachment site of the DDS is interior to the attachment site of the LDS in the cup of half-ring B1 and the rostral attachment site is at the robust bony ridge on the tympanum running parallel to the central axis of the syrinx (Figure 8G,H).

Second, the relatively large *musculus tracheobronchialis dorsalis *or dorsal tracheobronchial muscle (DTB) attaches caudally in the cup at the dorsal end of half-ring B2, and the rostral attachment site is not on bone but on the CASM near tracheal ring T3. Third, the *musculus tracheobronchialis brevis *or short tracheobronchial muscle (STB) attaches caudally on the lateral edge of the dorsal portion of half-ring B2 just outside the ventral cup. Some STB fibers also insert directly into the lateral labium (Figure 9D). Moving rostrally, the STB runs from dorsal to ventral on the lateral surface of the tympanum. Its rostral attachment site is on the tympanum just dorsal to the attachment site of the DVTB (Figure 8D,H).

Two syringeal muscles have one attachment site outside of the syrinx and are therefore traditionally referred to as extrinsic (versus intrinsic) syringeal muscles. First, the *musculus sternotrachealis *or sternotracheal muscle (ST) attaches on a lateral portion of tracheal ring T1 (Figure 8D). The ST runs through the clavicular air sac and may therefore be a unique example of a vertebrate muscle running through air inside the body. Distally, the ST attaches on a dorsally oriented protrusion of the sternum (see below). Second, the *musculus tracheolateralis *or tracheolateral muscle (TL) attaches onto the CASM near tracheal ring T4 (Figure 9C) and its muscle mass runs along the trachea towards the larynx. We did not investigate where the TL muscles fibers attach rostrally. The length of the trachea greatly exceeds the length of regular muscle fibers, suggesting that the TL consists of serially connected fibers or that its fibers span only several tracheal rings.

In summary, we found that syringeal muscles are organized as intriguing parallel sheets of fibers. We redefined the intrinsic syringeal muscles based on precise localization of their attachment sites. These results permit us to conclude that closely apposed syringeal muscles potentially cause differential actuations via distinct attachment sites.

### Implications for the motor actuation of syringeal elements

**Figure 10:**
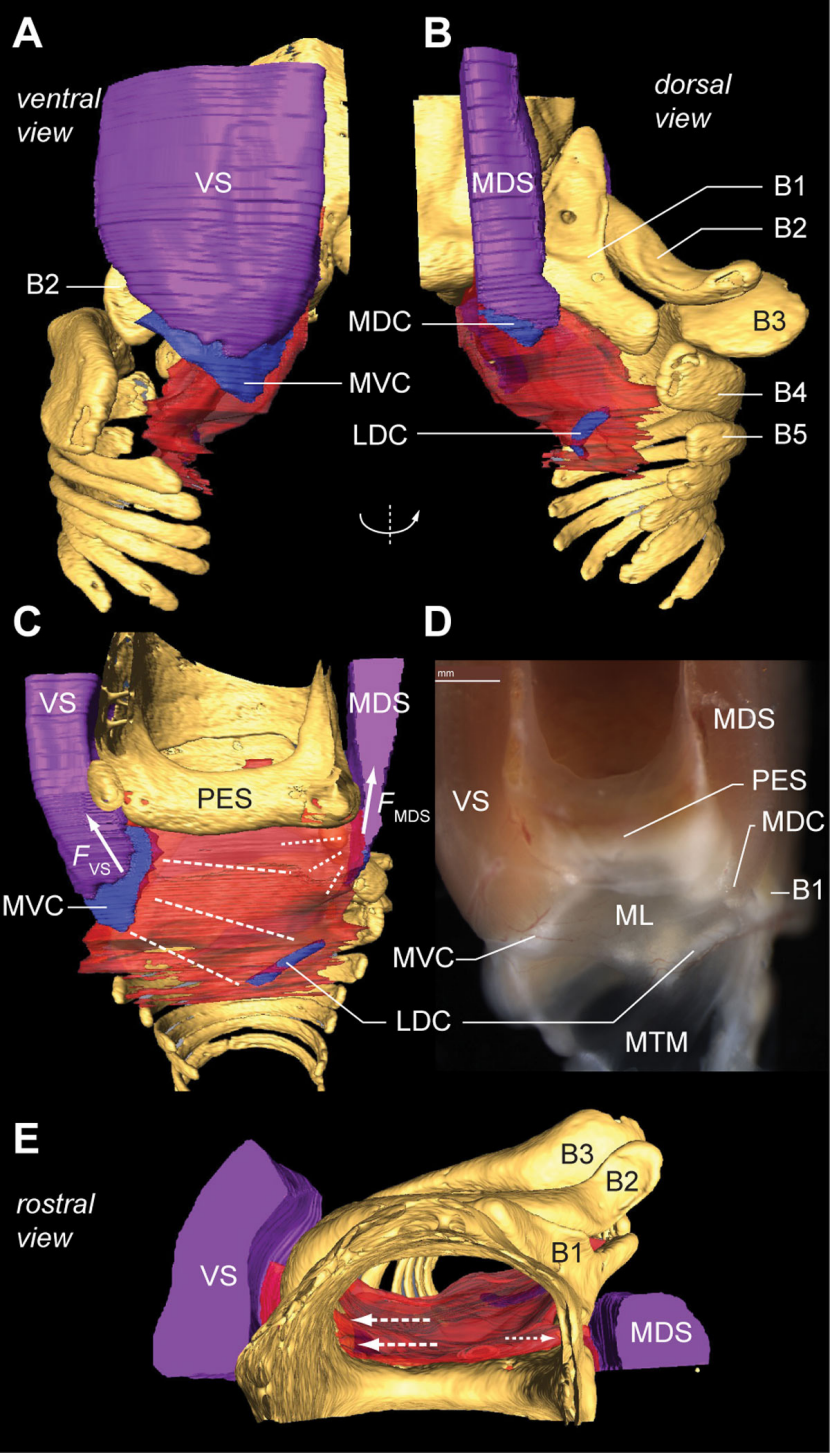
**Two syringeal muscles exert direct motor control on the medial vibratory mass**. **(A) **Ventral and **(B) **dorsal view of the right primary bronchus. The muscles VS and MDS insert directly on cartilaginous pads embedded in the MVM, hence controlling its tension. **(C) **Medial view of the MVM of the morphome and **(D) **of a fresh dissection. Contraction of the VS generates a force (*F*_VS_) that bends the MVC, thereby stretching (dotted lines) the ML towards the lateral dorsal cartilage (LDC). Energy stored within the MVC is released after VS contraction. This restores VS length back to resting length, and therefore also reduces the tension in the ML back to baseline. Contraction of the MDS generates a force (*F*_MDS_) that also increases tension in MVM, but with a different orientation. **(E) **The rostral view shows that the projected working lines (arrows) of the VS and MDS are aligned along the dorso-ventral axis. Contraction of these muscles therefore results in no or very little adduction of the ML into the bronchial lumen, and modulates tension only. Abbreviations as listed in Table 1. Colors as in previous figures.

The two bilateral pairs of labia are the principal sound generators in songbirds [24,26,30,32]. Our data show that two intrinsic syringeal muscles attach directly to cartilages that are embedded in the ML (Figure 10). Ventrally, the VS attaches to the MVC (Figure 8A) and, dorsally, the MDS attaches to the medial dorsal cartilage (Figure 10B). Because these cartilages are firmly embedded in the ML, our data suggest that, based on the orientation and location of the VS and MDS, shortening by either muscle would stretch the ML predominantly in the dorso-ventral axis. This would increase ML tension without changing ML ab- or adduction into the bronchial lumen (Figure 10C-E), which is consistent with earlier endoscopic observations during muscle micro-stimulation [23].

**Figure 11:**
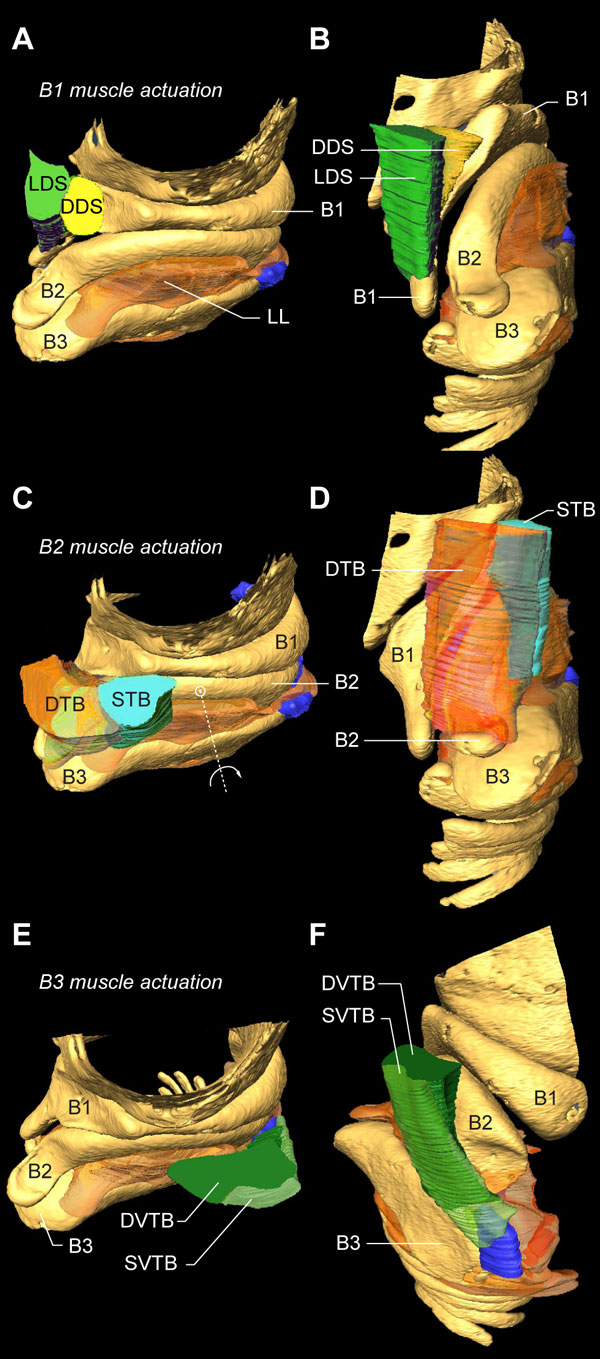
**Muscle actuation of bronchial half-rings B1-B3**. **(A) **Rostro-lateral and **(B) **dorso-lateral view of the right primary bronchus showing the muscles DDS and LDS that attach directly onto bronchial half-ring B1. **(C,D) **Same views as in A and B, but now showing the muscles DTB and STB that attach directly onto bronchial half-ring B2. The large VS muscle is omitted for clarity. **(E,F) **The muscles SVTB and DVTB attach directly onto bronchial half-ring B3. Abbreviations as listed in Table 1. Colors as in previous figures.

All other intrinsic syringeal muscles attach directly to bronchial half-rings B1 to B3 (Figure 11). Each half-ring has a parallel-running pair of muscles of which at least one muscle attaches to the tympanum and one more rostrally to the CASM. Two dorsal muscles actuate the bronchial half-ring B1: the LDS attaches to the tympanum and the DDS to the CASM (Figure 11A,B). Three muscles actuate the bronchial half-ring B2: on the dorsal side the parallel-running muscles DTB and STB (Figure 11C,D), of which the STB attaches to the tympanum and the DTB to the CASM. On the ventral side of the syrinx, some fibers of the VS attach to the ventral side of half-ring B2 (Figure 10A). Therefore both DTB and STB could act as functional antagonists to the VS, rotating half-ring B2 around a medial pivot (Figure 11C). Finally, two parts of the VTB actuate bronchial half-ring B3 of which the DVTB attaches to the tympanum and the SVTB to the CASM.

### *Position and stability of the zebra finch syrinx *in situ

**Figure 12:**
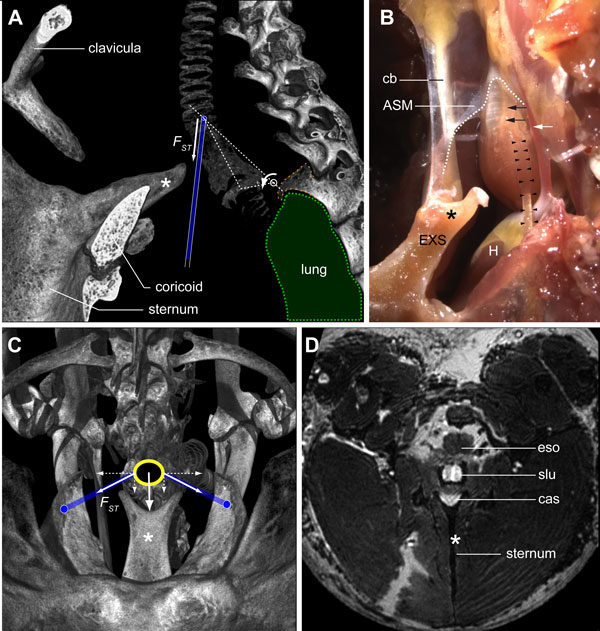
**The sternotracheal muscle stabilizes the syrinx during song**. **(A) **Lateral view of a μCT-based volume rendering showing the position of the syrinx in the skeletal framework of the upper thorax. A spine (orange dashed line) projects ventro-caudally from the second thoracic vertebra, thereby providing an anchor point for the lungs (green) and a pivot point (white circle) for the syrinx. On its ventral side, the syrinx fits into a dorsally oriented protrusion of the sternum, the external spine (EXS, *). The force (*F*_st_) exerted by contraction of the ST (blue) rotates the syrinx ventrally (arrow) into the external spine. **(B) **Lateral view of a dissected syrinx with the ST muscles (left ST, black arrowheads) and their attachments intact. The external spine is continuous with a collagenous band (CB) that connects to the CASM (dotted white line). Also visible are the *arteria syringealis*, which supplies blood to the syrinx (white arrow), and the left syringeal nerve (black arrows). **(C) **Caudal view of a μCT-based volume rendering looking up from the sternum showing the position of the syrinx (yellow circle) and the attachment sites of the ST muscles (blue lines) in the intact skeletal framework. The ST attaches to tracheal ring T1 and on two lateral protrusions of the sternum. Contraction of the ST muscles pulls the syrinx onto the EXS. **(D) **Virtual slice through a 3D MRI dataset showing the syrinx (yellow dotted line) and EXS. Abbreviations as listed in Table 1.

Our data support the hypothesis that shortening of the paired ST muscles can mechanically stabilize the syrinx during song [19,22,74]. Caudally to the syrinx, the primary bronchi are firmly anchored to the lungs, which are held in place against the vertebral column by a spiny intrusion of the second thoracic vertebra (Figure 12A). Micro-dissection shows that the syrinx is attached to this spine on the dorsal side by collagenous tissue. Furthermore, a thin, inflexible membrane, the interbronchial ligament (IBL), or bronchidesmus (Figures 8A and 9A), connects bronchial half-rings B4 and B5, thereby constraining rostral and lateral movement of the primary bronchi. These structures provide the caudal side of the syrinx with a rigid frame of reference relative to the spine (Figure 12A). In addition, on its dorsal side, the syrinx attaches to the esophagus and the vertebrae by collagenous sheets. We observed that ventral to the syrinx, the sternum protrudes into a Y-shape structure, the external spine (Figure 12). The ventral shape of the syrinx corresponds to the external spine (Figure 12B). Based on the orientation and attachment sites of the ST *in situ *(Figure 12C,D), our data suggest that co-activating the paired ST, leading to ST shortening, will pull the syrinx against the dorsal surface of the external spine, structurally stabilizing the syrinx relative to the skeleton.

## Discussion

As an essential prerequisite to understand how central song motor programs are translated into sound, we prepared a high-resolution annotated 3D morphome of the sound-producing organ, the syrinx, of the zebra finch (*T. guttata*), the principal avian model species for vocal learning. Using a multidisciplinary approach we identified and annotated in unprecedented detail hollow tracheo-bronchial bones, all syringeal muscles and their sheet architecture, a previously unrecognized Y-shaped structure on the sternum likely relevant for syringeal stabilization, and a cartilaginous structure in a position to enable uncoupling of sound frequency and amplitude control.

### Ossified structure of the zebra finch syrinx

Figures 2, 3 and 5 and Additional file 1 show that the structure of the syringeal skeleton is more complex than previously thought and inform us about the loads the skeleton experiences *in vivo*. The presence of hollow thin-walled bones in the syrinx (Figure 5) indicates an optimization of the skeleton through weight reduction and strength preservation [75]. Hollow cylindrical bones are advantageous because the bone's weight decreases with decreasing wall thickness while being able to resist equal bending moments without breaking [75]. In addition, lateral flattening of thin-walled bones further increases resistance to bending to perpendicular forces, because of increased second moment of area [76,77]. Thus bronchial half rings B1 to B3 provide high resistance to bending by forces (for example, $F_{⊥}$ in Figure 5D) perpendicular to the horizontal axis of their cross-section (indicated by the plane in Figure 5D). In addition, we found strut-like elements, trabeculae, that internally connect inner and outer walls. Bones this thin do not break but rather fail due to abrupt wall deformation, also known as buckling [75], and trabeculae prevent buckling. In constantly remodeling bone tissue [70], trabeculae indicate the need to prevent mechanical failure due to buckling, and therefore predict the direction of *in vivo *loads on the bone. In conclusion, the laterally flattened thin-walled hollow structure of the ossified bronchial half-rings of the zebra finch syrinx indicates that these bones are optimized for low weight while maintaining strength and are subjected to perpendicular loads *in vivo *[75-77].

The numerous trabeculae, especially in B1 and B3 (Figure 5B inset), prevent bending deformation, and as such strongly argue against bending of B1 and B2 as a mechanism for sound frequency modulation [43]. Their presence also indicates that high perpendicular forces are present. These can be attributed to extreme acceleration by superfast syringeal muscles, which can move syringeal elements cyclically up to 200 Hz and 250 Hz in zebra finch and starling males, respectively [36]. The absence of trabeculae in B2 suggests that the bending forces are lower here, which is most likely due to much slower force modulation by syringeal muscles.

We propose that the weight reduction of the syringeal skeleton and particularly of the bronchial half-rings B1, B2 and B3 is predominantly a result of physiological constraints on song production. In several songbirds species, females prefer song notes consisting of fast trills [78-83], suggestive of a selective pressure for the evolution of faster song elements that require faster movement of syringeal structures. The superfast muscles found in the songbird syrinx are pushed to the performance limits of vertebrate skeletal muscle so that their work output is 10 to 20 times lower than regular skeletal muscle [36,49,84] and a distinct trade-off exists between the work they can generate and the maximum frequency at which they can cyclically actuate objects [49,85,86]. Compared with the weight of a songbird (ranging from about 4.5 g (the goldcrest *Regulus regulus*) to 100 grams), the weight reduction of filled versus hollow syringeal bones (in the order of milligrams) must have very little effect on flight energetics. Lighter syringeal bones, however, can be moved at higher maximum speeds, enabling faster modulation and more precise temporal control of acoustic song features. Sexual selection pressure on syringeal performance therefore imposes additional costs on the syringeal skeleton, forcing it to adapt to avoid bone fracture.

### Cartilages of the zebra finch syrinx

We suggest that the medial ventral and medial dorsal cartilages embedded in the vibrating medial labia (Figures 7 and 10) are crucial to prevent tissue damage. They sum and transfer the forces produced by muscles to the labia in a graded manner. Instead, if the syringeal muscles VS and MDS (Figure 10) inserted directly on the ML, differential motor unit recruitment would result in large local strain discontinuities at the apex between two adjacent muscle fibers if only one fiber contracts. Without these cartilages distributing the applied forces evenly to the labia, the chance of severe tissue damage to the ML would be increased. The cartilages diffuse such large local strain discontinuities, a prerequisite for smooth modulation of tension.

Our data support the contention that the MVC of the syrinx also plays a crucial role in the mechanism to control sound frequency (Figure 10). Songbirds produce sound by flow-induced oscillations of the medial and lateral syringeal labia [24,26,30,32], which generate tracheal pressure fluctuations radiated as sound. Sound frequency seems to be controlled predominantly by muscles effecting tension changes in the medial, but not the lateral labium: VS activity correlates positively with fundamental frequency during song [21], and pulling on the MDS increases sound frequency *in vitro *[32]. By contrast, the amplitude (and gating) of sound appears to be predominantly controlled by muscles affecting the position of the lateral labium [22,23,36]. Our data therefore support the hypothesis that the primary mechanism controlling sound frequency by modulating ML tension is the bending of the flexible cartilaginous extension of bronchial half-ring B2, the MVC (Figure 10). Because bones easily break or buckle when bent, deformation of the half-rings B1 and B2 themselves [43] is not a suitable mechanism to modulate ML tension.

This MVC bending mechanism leads to two further hypotheses. First, energy stored in temporary elastic deformation of the MVC by VS shortening will act as a VS antagonist. Vertebrate striated muscle fibers need to be stretched back to resting length after contraction, which is often accomplished by contraction of an antagonistic muscle. In the zebra finch syrinx, release of the elastic energy stored within the MVC by VS contraction would restore the resting length of VS fibers and also the resting tension in the ML. Secondly, the MVC bending mechanism will enable the songbird syrinx to uncouple control of ML tension and position. In the mammalian larynx, tension in the vocal folds determines their oscillation frequency and, hence, the sound frequency produced, while the sound amplitude is determined by a combination of bronchial pressure and the amount of adduction of the vocal folds into the laryngeal lumen [87,88]. In two songbird species, the brown thrasher (*Toxostoma rufum *(Linnaeus, 1758)) and the northern cardinal (*Cardinalis cardinalis *(Linnaeus, 1758)), micro-stimulation of the VS bends the MVC and increases tension in the ML dorso-ventrally, but does not abduct the ML into the bronchial lumen [23]. We suggest that bronchial pressure (a result of respiratory muscle action) and the position of the lateral labium predominantly control sound amplitude in songbirds, whereas the tension in the ML locks the oscillation frequency for both labia and hence sets sound frequency. As such, the MVC is in a prime position to allow for the uncoupling of sound frequency and amplitude.

This uncoupling function of the MVC may have been key to the evolutionary success of songbirds or oscines (Passeriformes: Passeri). They first appeared about 55 million years ago [89] and now form the largest radiation within birds, including almost half of all 10,000 extant bird species [90]. Song has played a crucial role in the evolution of songbirds, driving sexual selection both intersexually (females choose mates based on song quality and male-male contests) and intrasexually (males use song to establish and defend territorial boundaries). Our comparative knowledge on song production is very limited. Most of our understanding of the physiology and different mechanisms of song production comes from a handful of species. The songbirds alone produce a wide variety of song, ranging from almost tonal to broadband sounds. This range of sounds might reflect differences in production mechanisms, syringeal tissue properties and morphology, as well as differential recruitment of the syringeal musculature.

Compared with the huge variation in syringeal morphologies found in non-songbirds, the morphology of the songbird syrinx is quite conserved [16,19,20]. Gaunt previously proposed that more elaborate intrinsic muscles in songbirds allowed for independent control of song parameters [20]. Field recordings [91], excised preparations [92], mechanical models [93,94] and numerical models of sound production in non-songbirds, such as ringdoves (*Streptopelia risoria*) [95], and sub-oscines (Passeriformes: Tyranni), such as the great kiskadee (*Pitangus sulphuratus*) [96], show that sound amplitude and frequency are often coupled to some degree, thereby severely limiting the potential repertoire of syllables that can be produced physically. To our knowledge, none of these species appears to have evolved a structure comparable to the MVC found in the songbird syrinx that could allow for tension control without affecting position. We speculate that the MVC contributed to the extensive diversification of songbirds because they evolved a syringeal morphology that allowed for largely independent control of sound parameters, opening up a wealth of song syllables not previously available.

### Implications for motor control

The morphome data (Figure 8 and Additional file 2) reveal that closely apposed muscles attach to different syringeal elements, which predicts distinct mechanical effects when recruited. In particular the dorsal musculature superficially looks like a continuous mass of muscle tissue (Figures 8 and 9, and Additional file 2), but our detailed analysis clearly demonstrates that spatially closely located fibers attach to (and therefore actuate) the ML, bronchial half-rings B1 and B2, and attach to six different locations on the tympanum and higher up the trachea in the CASM (Figures 8 and 9). The resulting subtle, but distinct, subdivision of syringeal muscles has significant implications for experiments addressing the function of specific muscles, because recording the activity or the individual stimulation of such physically closely located muscles is very sensitive to electrode design. The twisted fine-wire electrodes commonly used in syringeal electromyography (EMG) studies [21,37,97] and micro-stimulation studies [23,36,61] function as antennas that detect or cause cell membrane depolarization of many fibers and could easily cross boundaries of muscles with distinct biomechanical effects. The EMG signal strength depends upon wire diameter, impedance, length of stripped end and fiber size [98]. Because this effect is scale-dependent, using a species with a large syrinx partially circumvents this problem [21-23]. Nevertheless, one must be cautious when measuring and interpreting EMG recordings and micro-stimulation from a syrinx of a bird the size of the zebra finch.

Our *in situ *data (Figure 12) show that the syrinx can be lodged into the Y-shaped external spine of the sternum and therefore be stabilized with respect to the skeleton. This stabilization could reduce the amount of mechanical noise affecting the sound-producing labia, thereby augmenting the production of stereotyped songs. Several sources of mechanical noise are present during song. The beating of the heart and its major arteries provide rhythmic mechanical perturbation close to the labia and primary bronchi (Figures 1 and 12). During song, postural changes of the body due to courtship dances [99,100] affect the position of the trachea. To facilitate spectral changes of sound radiation, tracheal length is modulated by TL contraction [101] and changes in position of larynx and hyoid [38,40]. Additionally, the pressure of expired air from the lungs during vocalization can push the syrinx rostrally [23], and fast length modulations of the syringeal muscles can send vibrations up the trachea [74]. Co-contraction of the STs would press the syrinx onto the rigid framework that is composed of sternum, ribcage and spine.

EMG data support the idea that the ST is active during song [34].The ST has also been hypothesized to function as an adductor of the syringeal labia [99] and as an antagonist to the TL in many non-songbirds [95,102-108]. The ST muscle is present in all songbirds, and is missing only in very few bird species that are either non-vocal or that produce song with little frequency modulation (for example, the new world vultures [109,110] or Darwin's nothura (*Nothura darwinii *[111]). Smith [109] reported that surgical lesioning of the ST did not have much effect on acoustic song parameters in several songbird species. However, due to the very low sensitivity of the analysis possible at that time, this conclusion needs to be reassessed using modern quantification of sound parameters [54,56]. An important role for the ST in fine-tuning sound production is also implied, because of the high energetic costs [49] associated with superfast contractile properties as even found in ringdoves [97]. Furthermore, an activated ST can absorb vibration energy along the trachea by producing negative work when longitudinal vibrations are sent along the trachea due to fast shortening of intrinsic syringeal muscles [74].

The present refinement of muscle identity (Figure 8 and Additional file 2) also allows for more precise predictions about muscle co-activation known to increase accuracy and stability of position control in many complex motor systems such as arm reaching [112-114]. We currently do not know the recruitment patterns of all syringeal muscles simultaneously during song, but EMG recordings can demonstrate strong correlation between muscles [21]. With antagonistic control of a specific parameter, such as lateral labium position, different levels of recruitment of at least four relevant muscles (DTB, STB, DVTB, SVTB; Figure 11) could produce the same position of the lateral labium. Therefore different motor programs may result in similar acoustic features and, conversely, combinations of muscle activation, or synergies [65-67], might be required to achieve control values. If muscles are activated synergistically, it is not individual muscle activation but the underlying synergy that functionally correlates to sound parameters. This would imply that we should not aim to correlate sound parameters to individual muscle activity, but to underlying muscle synergies instead.

The morphome presented here provides a crucial foundation for mapping motor projections of the song system onto the syringeal muscles and to study how syringeal muscle recruitment shapes sound production. To ultimately assess muscle function during song production, muscle stimulation and perturbation experiments in controlled environments and during song need to be performed. Such studies will ultimately link syringeal biomechanics (that is, the determination of pivoting points and mechanical properties of the bronchial half-rings, their mechanical coupling and kinetics) to sound and song parameters. In fact, it is impossible to meaningfully connect the song system at the level of hindbrain motoneurons to the syrinx without a thorough understanding of the number, orientation and function of syringeal muscles. Additionally, such a motor map will allow the translation of commonly used sound parameters [53,54,56] to neural firing patterns. The syringeal geometry presented here provides a quantitative basis for implementing realistic 3D morphologies into computational models of sound production. The parallels between birdsong and human speech observed at the behavioral, neural [2,10,115] and genetic level [116] are incomplete until the peripheral contribution to human speech and birdsong are understood at a similar level of detail. To grasp how central motor patterns are translated into vocal communication, it is important to understand peripheral mechanisms of sound production across the vocal vertebrates.

## Conclusions

The avian vocal organ, the syrinx, is a key component in the hierarchy of structures responsible for translating central song motor programs into acoustic behavior. This study presents the first high-resolution annotated dataset, or morphome, containing the 3D structural organization of the zebra finch syrinx *in situ*, based on results derived from MRI, μCT, histology and micro-dissection. The zebra finch syrinx morphome provides the substrate for an understanding of how the neural code is translated into the combined action of the individual components of the musculoskeletal apparatus. It provides comprehensive insights into syringeal structure and function with important implications for muscular control, and sheds light on the evolution of signaling systems in birds.

## Methods

### Animal use and care

Male and female zebra finches (*T. guttata *(Vieillot, 1817); Aves: Passeriformes: Estrildidae) were kept in group aviaries at the Freie Universität, Berlin, Germany and the University of Southern Denmark, Odense, Denmark on a 12 h light:dark photoperiod and given water and food *ad libitum*. All procedures were carried out in accordance with the Landesamt für Gesundheit und Soziales (Berlin, Germany) and the Danish Animal Experiments Inspectorate (Copenhagen, Denmark).

### Magnetic resonance imaging

Zebra finches (six males, six females) were deeply anesthetized with isoflurane and transcardially perfused with heparinized 0.1 M PBS followed by 4% phosphate-buffered paraformaldehyde (PFA). We performed MRI scans of whole animals (one male: one female) at the Institut für Klinische Radiologie in Münster, Germany using a Bruker Biospec 94/20, 9.4 T, high-field, horizontal-bore, small animal scanner (Bruker BioSpin GmbH, Ettlingen, Germany). MRI scans of dissected syrinxes were performed at the Institut für Experimentelle Physik 5 in Würzburg, Germany using a Bruker Avance 750WB, 17.6 T, high-field, vertical-bore, small animal NMR scanner equipped for imaging (Bruker BioSpin GmbH). Intact animals were scanned in PBS with 2 mM gadopentetate dimeglumine (Magnevist; BayerSchering GmbH, Berlin, Germany) as contrast agent. We used the following scanning parameters: Turbo RARE 3D protocol, 680 × 296 × 256 pixel matrix size, 6.5 × 2.85 × 2.5 cm field of view, 96 × 96 × 98 μm voxel resolution, T_R _= 1500 ms, T_E _= 14.7 ms, eight averages, RARE factor 16, and 15 h 21 min 36 s acquisition time. We additionally scanned the head of one male zebra finch using the following parameters: Turbo RARE 3D protocol, 288 × 288 × 256 pixel matrix size, 25.88 × 25.88 × 23.00 mm field of view, 90 μm isotropic voxel resolution, T_R _= 1500 ms, T_E _= 9.97 ms, eight averages, RARE factor 16, and 15 h 21 min 36 s acquisition time.

In addition, we scanned excised syrinxes (five males, five females). After fixation as described above, syrinxes were removed, post-fixed in 4% PFA overnight, and stored at -20°C in 30% sucrose with 0.01% sodium azide. For mechanical stabilization, we embedded the preparations into low-melting agarose (Carl Roth, Karlsruhe, Germany) with 0.01% sodium azide and 2 mM Magnevist as contrast agent. We then placed them inside 15 ml Falcon tubes (15 mm diameter) for scanning using the following scanning parameters: FLASH 3D protocol, 256 × 290 × 290 to 512 × 512 × 512 pixel matrix size, 1.2 × 1.2 × 1.2 to 1.4 × 1.4 × 1.4 cm field of view, 23 to 46 μm isotropic voxel resolution, T_R _= 20 to 22 ms, T_E _= 3.3 to 5.4 ms, 9 to 32 averages, and 3 h 38 min 27 s to 18 h 0 min 32 s acquisition time.

All MRI datasets were analyzed using the Volume Viewer plugin in ImageJ (NIH, Bethesda, MD, USA).

### Micro-computed tomography

We performed whole animal μCT scans (one male, three females) at the Museum für Naturkunde (Berlin, Germany) using a Phoenix Nanotom X-ray tube tomography system with a 2,300 × 2,300 pixel detector (GE Sensing & Inspection Technologies, Wunstorf, Germany). Animals were overdosed with isoflurane and placed inside air-filled 50 ml Falcon tubes after respiration had stopped for 5 min. The following scanning parameters were used: 150 to 180 kV source voltage, 145 to 270 μA source current, no filter, 750 ms exposure time, 13 to 22 μm isotropic voxel resolution, 1,440 angular steps over 360° with three averaged images per rotation position, and about 54 min scan time.

We also made scans of isolated syrinxes (12 males, 5 females) to increase scan resolution with respect to whole animal scans. These scans were performed at the Center for Nanoscale Systems (Cambridge, MA, USA) using a MCT 225 X-ray tube tomography system with a 2,000 × 2,000 pixel detector (Nikon Metrology, Leuven, Belgium). Both μCT scanners used in this study were equipped with a tungsten target. Specimens were overdosed with isoflurane and the syrinxes were dissected with a large portion of esophagus, trachea and lungs attached to maintain structural integrity. We took special care to mount the preparations in their natural position onto 3 mm thick silicone sheets using 0.1 mm diameter stainless steel Austerlitz insect pins (Fine Science Tools, Heidelberg, Germany). The samples were fixed for seven days in 4% PFA in PBS stored at 4°C. After removal of the needles, the samples were transferred to 1 ml pipette tips filled with PBS and stored at 4°C until scanning.

We made two scans of each preparation. First, we optimized scanning parameters to assess the anatomy of the ossified syringeal skeleton. We scanned the samples using the following parameters: 85 kV source voltage, 110 μA source current, 0.1 mm copper filter, 2 s exposure time, 5 μm isotropic voxel resolution, 1,440 angular steps over 360° with two averaged images per rotation position, and about 1 h 36 min scan time. Second, we added contrast agent and optimized scanning parameters to image soft tissues. We stained the samples inside glass vials with 0.1% Lugol's solution (aqueous I_2_/KI, Sigma Aldrich, St. Louis, MO, USA) and placed them on a tube roller for 48 h following previous protocols [117-119]. Prior to scanning, the samples were rinsed in distilled water for 10 min twice and then placed inside 1 ml pipette tips with distilled water. We scanned the samples using the following parameters: 85 kV source voltage, 110 μA source current, 0.1 mm copper filter, 2 s exposure time, 5 μm isotropic voxel resolution, 1,440 angular steps over 360° with two averaged images per rotation position, and about 1 h 36 min scan time. Dataset reconstruction was performed using the software provided with each scanner, that is, Phoenix DatosX Reconstruction 1.5 for the GE system and Metris XT 2.2 for the Nikon system. Reconstruction was performed with noise reduction activated, but without binning.

We quantified shrinkage by measuring the distance between the IBL and the top of tracheal ring T2 from photographs of the preparations taken immediately after dissection and after seven days of PFA fixation. Shrinkage averaged 1.3% and was less than 4.8% in all preparations.

### Fresh tissue micro-dissection

We performed micro-dissection (six males, five females) after euthanasia by isoflurane overdose. The syrinxes were exposed by cutting through the pectoral muscles and sternum. They were carefully isolated and pinned down on Sylgard-covered Petri dishes in oxygenated Ringers solution (NaCl, 154 mM; glucose, 12 mM; KCl, 6 mM; MgCl_2_, 1 mM; NaH_2_PO_4_, 1 mM; MgSO_4_, 1 mM; HEPES, 10 mM; CaCl_2_, 4 mM; adjusted to pH 7.4 with Trizma Base) at room temperature (18°C). Previous muscle mechanics experiments on syringeal muscles have shown that the muscles retain contractile abilities for up to 8 hours under these conditions [36,74,97]. The syrinx was separated medially into left and right hemisyrinxes that were separately stored in oxygenated Ringers solution either on icepacks or at room temperature (18°C). We studied syringeal tissues by careful micro-dissection under a M165-FC stereomicroscope (Leica Microsystems, Wetzlar, Germany) using a CLS-150XE light source (Leica Microsystems). Images were taken with a DFC425-F camera (Leica Microsystems). Muscle fibers were visualized using variable lighting conditions to achieve large contrast. Attachment sites of each muscle were photographed and noted on various 2D projections of previously generated μCT-based 3D volume renderings of the syringeal skeleton.

### Histology

For histological examinations, we perfusion-fixed the syrinxes of six animals (four males, two females) in 4% PFA and decalcified them in a mixture of 4% PFA/ethylenediaminetetraacetic acid for one week. We then dehydrated them in an ascending alcohol series of 30%, 50% and 70% ethanol for 10 min per step on an automatic shaker to facilitate tissue penetration. The ethanol was gradually replaced with paraffin using a TP 1020 tissue processor (Leica Microsystems). Finally, syrinxes were embedded in melted paraffin in an aluminum tube covered with a perforated plastic cap. The embedded syrinxes were cooled down overnight to about -20°C. A rotary microtome (Microm HM 340e, Thermo Scientific, Billerica, MA, USA) was used to section the syrinxes into 10 μm thick sections. Sections were stained using Mayer's hematoxylin and eosin staining protocol and examined under a MDG30 microscope (Leica Microsystems). A DFC420C camera (Leica Microsystems) was used for imaging.

### Morphome annotation

Out of all the μCT-based syringeal datasets, we chose one representative male and female specimen, based on optimal tissue contrast, presence of all structures and correspondence to *in vivo *orientation. All recognized muscles, muscle attachment sites, cartilaginous pads and extensions, and medial and lateral labia were annotated onto this dataset for the male and female syrinx. Over 10 different systems of muscle nomenclature exist to describe syringeal muscles in songbirds [19]. Ames [19] studied the syrinx of 533 songbird species and concluded that the different nomenclatural systems were divided by two main issues: '1) whether muscles should be named based on the basis of function or on location, and 2) if a single mass of muscle fibers attached to several cartilaginous elements constitutes one muscle or more'. Because of insufficient data on syringeal muscle function, we decided to use a combined approach to nomenclature, based first on location (*sensu *Owen [120]). Secondly, working towards a functional nomenclature, we considered distinct attachment sites to define muscles, because the recruitment of muscles with different attachment sites is the basis of differential actuation of syringeal elements, which in turn will lead to differential biomechanical effects on sound production.

The syrinx morphome was annotated in consensus, based on comparative analysis of histology, μCT scan projections, 3D models and micro-dissection and additional micro-dissections of two males and two females at the University of Southern Denmark, Odense.

### Analysis software

3D volume and surface rendering and visualization were performed using myVGL 2.1 (Volume Graphics GmbH, Heidelberg, Germany), Amira 5.2 (Visage Imaging GmbH, Berlin, Germany), and ImageJ and its Volume Viewer plugin (NIH). All morphome annotation and data analyses were performed using Amira. The interactive 3D PDF models were created using Adobe Acrobat 9.4 and Adobe 3D Reviewer 9.4 (Adobe Systems, San Jose, CA, USA) following procedures described elsewhere [68,69].

## Abbreviations

2D: two-dimensional; 3D: three-dimensional; EMG: electromyography; MRI: magnetic resonance imaging; PBS: phosphate-buffered saline; PFA: phosphate-buffered paraformaldehyde; μCT: micro-computed tomography. For anatomical nomenclature, see Table 1.

## Competing interests

The authors declare that they have no competing interests.

## Authors' contributions

DND, AlZ, CKT, AnZ, CS and CPHE conceived of the study. DND, AlZ, CKT, CF, JM and CPHE contributed to data acquisition. AlZ, AnZ, JM, CF, CS and CPHE contributed reagents, materials and analysis tools. DND, AlZ, CKT, CF, JM, CS and CPHE contributed to data analysis. DND, AlZ, CKT, CS and CPHE wrote the manuscript. All authors read and approved the final manuscript.

## Supplementary Material

Additional file 1 **Syringeal skeleton of the male and female zebra finch syrinx (interactive 3D PDF)**. This 3D PDF figure can be viewed with Adobe Acrobat Reader version 9 or higher (http://www.adobe.com). Click on the figure to activate the 3D features. Tyzack [121] provides a comprehensive introduction to the use of interactive 3D PDF files, while Kumar and colleagues [69,122] provide instructions on how to create interactive 3D PDF files. Click here for file

Additional file 2 **Zebra finch male syrinx morphome (interactive 3D PDF)**. This 3D PDF figure can be viewed with Adobe Acrobat Reader version 9 or higher (http://www.adobe.com). Click on the figure to activate the 3D features. Tyzack [121] provides a comprehensive introduction to the use of interactive 3D PDF files, while Kumar and colleagues [69,122] provide instructions on how to create interactive 3D PDF files. To keep the PDF size of the morphome manageable, the size of the model had to be reduced. Therefore, very thin parts such as muscle insertions and the ends of muscles do not always appear to be correctly placed. Please refer to text and figures for exact descriptions. Click here for file
