# Supplementary figures and images for: The songbird syrinx morphome: a three-dimensional, high-resolution, interactive morphological map of the zebra finch vocal organ
Source: BMC Biol. 2013 Jan 8;11:1. doi: 10.1186/1741-7007-11-1 (PMC3539882; doi:10.1186/1741-7007-11-1)

♂

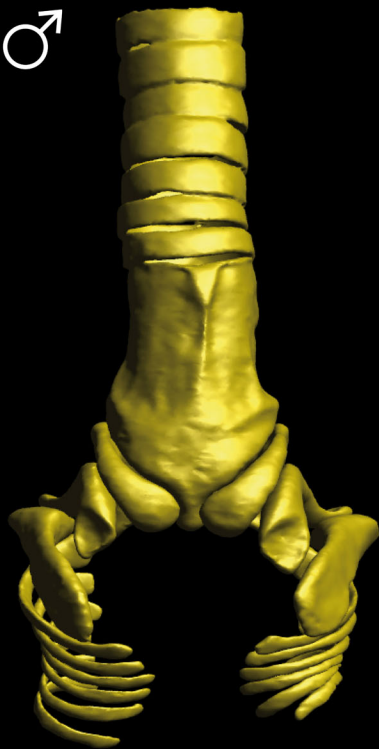

♀

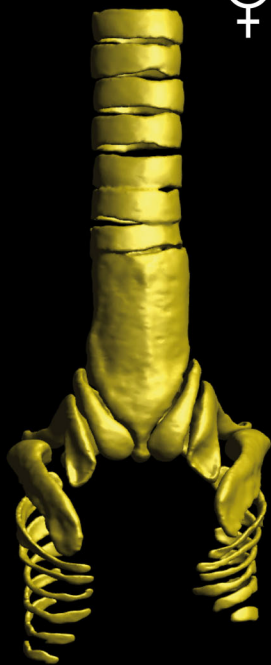

1 mm

Supplement: Additional file 1 — Syringeal skeleton of the male and female zebra finch syrinx (interactive 3D PDF). This 3D PDF figure can be viewed with Adobe Acrobat Reader version 9 or higher (http://www.adobe.com). Click on the figure to activate the 3D features. Tyzack [121] provides a comprehensive introduction to the use of interactive 3D PDF files, while Kumar and colleagues [69,122] provide instructions on how to create interactive 3D PDF files. [file 1741-7007-11-1-S1.PDF]

♂

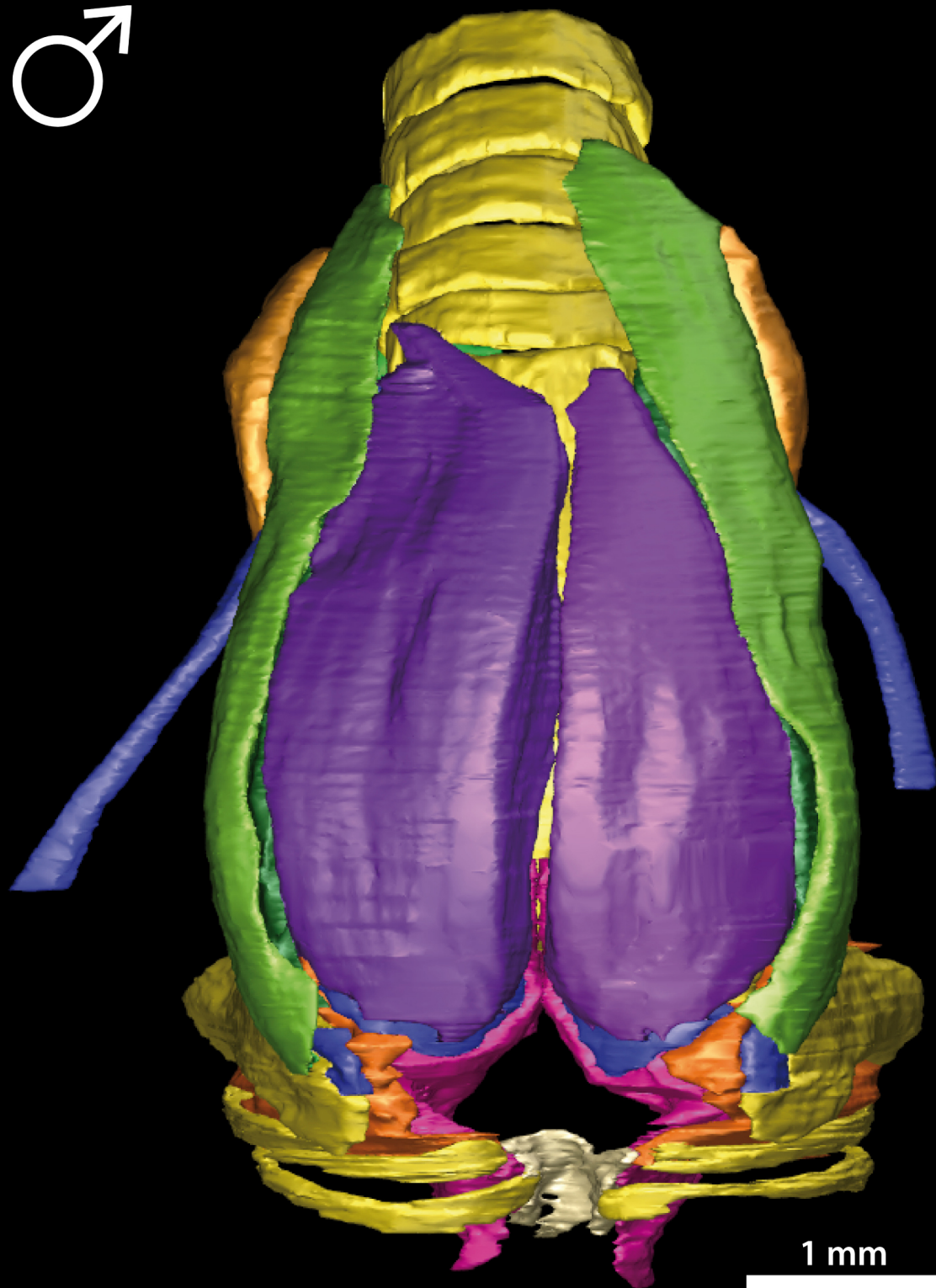

1 mm

Supplement: Additional file 2 — Zebra finch male syrinx morphome (interactive 3D PDF). This 3D PDF figure can be viewed with Adobe Acrobat Reader version 9 or higher (http://www.adobe.com). Click on the figure to activate the 3D features. Tyzack [121] provides a comprehensive introduction to the use of interactive 3D PDF files, while Kumar and colleagues [69,122] provide instructions on how to create interactive 3D PDF files. To keep the PDF size of the morphome manageable, the size of the model had to be reduced. Therefore, very thin parts such as muscle insertions and the ends of muscles do not always appear to be correctly placed. Please refer to text and figures for exact descriptions. [file 1741-7007-11-1-S2.PDF]
